# Inverted Classroom Teaching of Physiology in Basic Medical Education: Bibliometric Visual Analysis

**DOI:** 10.2196/52224

**Published:** 2024-06-25

**Authors:** Zonglin He, Botao Zhou, Haixiao Feng, Jian Bai, Yuechun Wang

**Affiliations:** 1 School of Basic Medicine and Public Health, Jinan University, Guangzhou, China; 2 Division of Life Science, Hong Kong University of Science and Technology, Clear Water Bay, Kowloon, HKSAR, China; 3 Gies College of Business, University of Illinois Urbana-Champaign, Urbana-Champaign, IL, United States; 4 School of Education, South China Normal University, Guangzhou, China

**Keywords:** flipped classroom, flipped classroom teaching, physiology, scientific knowledge map, hot topics, frontier progress, evolution trend, classroom-based, bibliometric visual analysis, bibliometric, visual analysis, medical education, teaching method, bibliometric analysis, visualization tool, academic, academic community, inverted classroom

## Abstract

**Background:**

Over the last decade, there has been growing interest in inverted classroom teaching (ICT) and its various forms within the education sector. Physiology is a core course that bridges basic and clinical medicine, and ICT in physiology has been sporadically practiced to different extents globally. However, students’ and teachers’ responses and feedback to ICT in physiology are diverse, and the effectiveness of a modified ICT model integrated into regular teaching practice in physiology courses is difficult to assess objectively and quantitatively.

**Objective:**

This study aimed to explore the current status and development direction of ICT in physiology in basic medical education using bibliometric visual analysis of the related literature.

**Methods:**

A bibliometric analysis of the ICT-related literature in physiology published between 2000 and 2023 was performed using CiteSpace, a bibliometric visualization tool, based on the Web of Science database. Moreover, an in-depth review was performed to summarize the application of ICT in physiology courses worldwide, along with identification of research hot spots and development trends.

**Results:**

A total of 42 studies were included for this bibliometric analysis, with the year 2013 marking the commencement of the field. University staff and doctors working at affiliated hospitals represent the core authors of this field, with several research teams forming cooperative relationships and developing research networks. The development of ICT in physiology could be divided into several stages: the introduction stage (2013‐2014), extensive practice stage (2015‐2019), and modification and growth stage (2020‐2023). Gopalan C is the author with the highest citation count of 5 cited publications and has published 14 relevant papers since 2016, with a significant surge from 2019 to 2022. Author collaboration is generally limited in this field, and most academic work has been conducted in independent teams, with minimal cross-team communication. Authors from the United States published the highest number of papers related to ICT in physiology (18 in total, accounting for over 43% of the total papers), and their intermediary centrality was 0.24, indicating strong connections both within the country and internationally. Chinese authors ranked second, publishing 8 papers in the field, although their intermediary centrality was only 0.02, suggesting limited international influence and lower overall research quality. The topics of ICT in physiology research have been multifaceted, covering active learning, autonomous learning, student performance, teaching effect, blended teaching, and others.

**Conclusions:**

This bibliometric analysis and literature review provides a comprehensive overview of the history, development process, and future direction of the field of ICT in physiology. These findings can help to strengthen academic exchange and cooperation internationally, while promoting the diversification and effectiveness of ICT in physiology through building academic communities to jointly train emerging medical talents.

## Introduction

In recent decades, student-centered active learning strategies have been implemented in numerous educational institutions worldwide as an alternative to traditional passive learning strategies such as didactic lecturing [1]. As a novel teaching mode, inverted classroom teaching (ICT), first proposed by Lage et al [2] in 2020, is now widely used to enhance the engagement of students in the active learning process. ICT, also known as “flipped classroom teaching,” promotes student participation, engagement, and identification of necessary resources and needs to meet learning objectives by repurposing classroom time for student-centered learning activities [3,4]. The teaching materials are made available for self-study outside of the classroom, while ICT also emphasizes active learning by assigning preclass tasks to students with clear learning objectives. ICT represents a significant advancement in modern classroom design, and its potential for promoting student-centered learning is particularly noteworthy.

Medical institutions were among the first to shift away from traditional didactic methods toward student-centered learning, which has been shown to motivate and empower students to be life-long learners, foster self-growth, and encourage receiving and applying up-to-date information and techniques in various medical fields [5,6]. Since it was first proposed as a teaching model [2], ICT has been used in almost all fields of education, especially in basic medicine and clinical medicine, and has become a focus of educational research. A recent bibliometric analysis on ICT revealed its ability to reallocate the teaching content taught in traditional classrooms outside the classroom for students to study on their own before the class. The resulting saved classroom time is then used for various student-centered learning activities such as problem-based and inquiry-based learning [4,7,8]. With the COVID-19 pandemic wreaking havoc around the globe, ICT has been increasingly incorporated into online teaching and is regarded as a promising and flexible approach for securing high-quality teaching via different forms of teaching media [9]. Despite the overwhelming benefits and compelling cases, researchers have also reported negative examples and disadvantages of using active-learning strategies, such as students lacking learning motivation [10,11], increased workload for both faculty and students [12], longer preparation time [12], and reluctance to discuss the teaching content with peers [13]. Moreover, a systematic theoretical and practical system of ICT in medical education has not yet been established.

Physiology is a bridging course between basic and clinical medicine, which is a core course for students in medicine and related subjects. Physiology is typically scheduled in the first semester of the second year of medical school. This course is often considered challenging for students in the early stages of their medical education owing to its highly conceptual nature, the significant cognitive effort required to acquire academic information, and the combined laboratory experiments associated with theoretical knowledge [9,14,15]. To a certain extent, the history and development of inverted teaching in physiology may serve as a window to probe into the general picture of the use of ICT in basic medical education. However, there is still a vast knowledge gap in the development and application of ICT in physiology courses; for example, it remains unclear how ICT in physiology evolved from the information era to the digital and artificial intelligence era. With the development of CiteSpace, a powerful visualization and analysis software, it has now become feasible to depict and visualize science knowledge graphs [16], including the outline and timeline of ICT in physiology, which can help to address these knowledge gaps in a more quantitative manner than possible with traditional qualitative methods such as a scoping review.

Therefore, in this study, we performed a visual analysis of the ICT in physiology literature from the Web of Science (WoS) database with CiteSpace. The aim was to explore the temporal evolution context and spatial distribution networks of ICT in physiology; investigate the cooperation network among authors, institutions, and countries publishing research in this field using co-occurrence analysis; and uncover hot research topics and development trends through cocitation analysis of references, authors, and journals, along with keyword co-occurrence and clustering analyses.

## Methods

### Search Strategy

We selected the WoS Core Collection as the data source for this study. To capture a broad range of potentially eligible articles, we used the following search terms with Boolean operators: (“flipped classroom” OR “flipped classroom teaching” OR “flipped study” OR “flipped learning” OR “flipped teaching” OR “flipped instruction” OR “inverted teaching” OR “inverted learning” OR “inverted study” or “inverted classroom” OR “inverted instruction”) in all fields AND (“Physiology”) in all fields. The time span was set from January 2000 to April 2023, and the data were collected on December 11, 2023. Only journal articles indexed in the WoS Core Collection were used to gather data. This database was selected because it is the longest-established citation tracking database, which includes quality indices such as Journal Citation Reports [17], provides a well-recognized subject classification for research journals, and permits the easy download of a considerable number of stored references [18].

### Study Selection Criteria

The search was performed in English to obtain the largest number of documents in the WoS data set on the use of ICT in physiology education. The following inclusion criteria were applied: (1) document type=articles, (2) language=English, (3) years of publication=2000-2023 (November). The exclusion criteria were (1) studies in a field not related to medicine or pedagogy; (2) not published in English; (3) categorized as books, chapters, theses, protocols, study outlines, government publications, posters, editorial materials, duplicates, or nonpeer-reviewed articles; and (4) published outside of the time frame of 2000-2023.

Upon applying the above search strategy, 632 indices were retrieved in the WoS data set and 295 records were screened after removing 237 studies using automation tools from the database. Before further screening and retrieval of the full texts of the references, all 294 indices with detailed citation records and bibliometric information were exported in both record and reference formats, saved as plain-text files, and stored in the .txt format. The stored records were then input into the CiteSpace software for visualization, as indicated by the user manual [19], which generated clustered plots of bibliometric references and differentiated various topics. The relevant articles pertaining to inverted classroom pedagogy were identified by examining the visualized clusters and topics, and irrelevant literature was excluded by adhering to the guidelines in the CiteSpace manual. In brief, in the cluster plots, irrelevant topics are presented in isolated clusters without citation networks; hence, these dots, representing the irrelevant literature, were removed from the eligible references after reviewing the titles and abstracts.

The full text of the included articles was downloaded and reviewed by two authors independently (YW and ZH), and a consensus was reached through discussion between the two reviewers in the case of any disagreements. In total, 253 studies were excluded after title and abstract screening and a total of 42 articles were included for the final analysis. The flowchart of study selection is provided in Multimedia Appendix 1 and the details of the excluded studies with reasons for exclusion are provided in Multimedia Appendix 2.

### Data Analysis Process

CiteSpace 6.1.R6 software was used to visually analyze the literature related to ICT in physiology published up to November 2023. CiteSpace is a knowledge visualization software developed by Chaomei Chen at Drexel University and is now a widely used knowledge mapping tool in various fields of education and teaching [20]. CiteSpace can measure and visualize literature collections in broad fields of the natural and social sciences using cocitations of references, authors, and journals; the co-occurrence of authors, keywords, institutes, and countries; and cluster analysis to create a scientific knowledge network map, explore the critical path of the evolution of the discipline, and analyze the hot spot research topics and frontier trends clearly and scientifically.

In this study, we analyzed the overall national and regional distributions and cooperation of the authors of ICT in physiology research papers through the constructed network cooperation map, and then determined the knowledge base and the core authors of ICT in physiology research through analysis of the literature and author cocitation networks. We further identified the “star” journals publishing research in this field through a cocitation analysis of the source journals. Finally, the hot spot keywords were determined through keyword co-occurrence and clustering analysis based on the frequency and centrality of the keywords, which were used to further explore the hot topics of worldwide research on ICT in physiology. Overall, the methodology used in this study involved cooperative network analysis and cocitation analysis.

Cooperative network analysis was used to identify core authors, leading research institutions, and national/regional cooperation in ICT in physiology research. The nodes in the graph are represented by circles, with larger circles indicating a greater number of items represented, such as papers, authors, institutions, references, and countries. In CiteSpace, intermediary or between centrality is used as a critical indicator of node importance, which is characterized by the shortest number of paths passing through a node. Nodes with a centrality value above 0.1 are considered to be important. In this study, the circle size represents the cited frequency of an article, with purple circles indicating high centrality; thus, larger and deeper-purple circles suggest greater importance of the study in ICT in physiology research.

Cocitation analysis was used to identify relationships between cited articles, authors, and journals in the field of ICT in physiology research. For example, if two articles (or authors or journals) A and B are cited simultaneously by a third article, then a cocitation relationship exists between them. Frequent citation of articles (or authors or journals) together suggests that their research topics, including concepts, theories, or methods, are likely related. Cocitation analysis ranks key papers according to their citation frequency and explains the correlation between their contents and directions through the centrality value. This analysis can also infer literature clusters from various papers that are published during the same period, indicating hot spots in the field. The frequency and relevance of citations represent hot spots in scientific research over time, and these core documents form the knowledge base for the hot spots. In turn, the knowledge base clarifies the cutting-edge nature of the research, as frequently cited papers constitute the corresponding knowledge base [21].

## Results

### Publication Trends in ICT in Physiology

The year 2013 marks the commencement of the field, in which Tune et al [22] were the first to publish a research paper related to ICT in physiology. The research volume then increased yearly, reaching its peak in 2022. According to the number of publications, different stages of ICT in physiology development can be defined. Before 2017, there were only a small number of papers related to ICT in physiology, marking 2013‐2017 as the gradual upward stage. In 2018, there was a slight decrease in the number of published papers on the topic, which may be due to the conflicts between conventional teaching and incorporating ICT into physiology teaching, indicating the need for more modification and reflection in practice. Hence, 2018‐2019 can be considered as the adaptation period. The second gradual upward period appeared during the COVID-19 outbreak in 2021 and then peaked in 2022, indicating a boom period for this field of study.

### Authors’ Cooperative Network

An author’s contribution to the area of ICT in physiology can be identified by their significant publications and cooperative connections with other authors, which facilitates understanding the progress in ICT in physiology [23]. Author collaboration appears to be generally limited, and most academic work in this field is conducted in independent teams with minimal cross-team communication.

**Figure 1.**
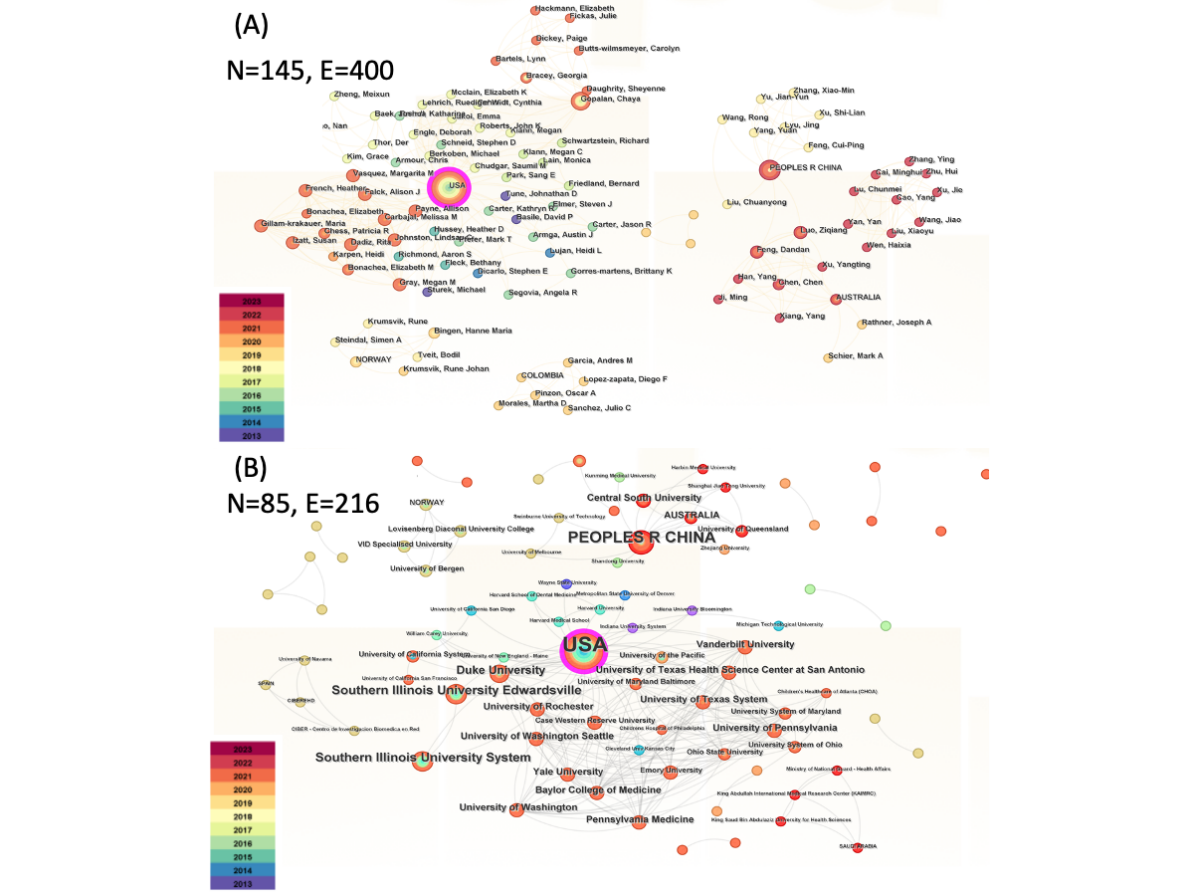
Network analysis map. The collaboration networks for (**A**) authors and national/regional collaboration (N=145, E=400) and (**B**) institutions (N=85, E=216) in the field of inverted teaching in physiology. Node size (N) corresponds to the frequency of inverted teaching in physiology publications from each author/institution. The connecting lines (E) represent collaborative connections between authors/institutions, with thicker lines indicating more frequent collaboration.

As shown in Figure 1A, the research author cooperation map highlights various research partnership teams, particularly those surrounding the authors Gopalan C, Gillam-Krakauer M, and multiple researchers with cooperative connections. Gopalan C has the highest citation count with 5 publications, followed by authors Carbajal MM, Falck AJ, Johnston LC, Feng D, Luo Z, French H, Dadiz R, Vasquez MM, and Gray MM who collaborated on three records with a citation count of 3 each, as depicted in Multimedia Appendix 3.

Since 2016, Gopalan C has published 14 relevant papers, with a significant surge from 2019 to 2022, as illustrated in Figure 1A. Gopalan C, Bingen H, Tveit B, Steindal S, and Krumsvik R have jointly published three papers centered on nursing education [24-26], indicating a stable partnership among these authors who conducted a series of studies on ICT in nursing education. Additionally, some other authors, including Feng D and Luo Z from Central South University in China, have coauthored two papers [27,28].

### National/Regional and Institutional Cooperative Networks

Overall, the extent of collaboration between nations and research institutions is relatively weak, with very low centrality, and the research power of countries is uneven. As seen in Figure 1 and Multimedia Appendix 4, US-based authors published the highest number of inverted teaching in phyisology–related papers (18 in total, accounting for over 43% of the total papers). Moreover, their intermediary centrality is 0.24, indicating that they have strong connections and are highly engaged in international cooperation. Chinese authors ranked second, publishing 8 papers; however, their intermediary centrality is only 0.02, suggesting that their papers have limited international influence and lower overall quality, providing little influential power in the field of ICT in physiology. Australia ranked third, with a centrality of 0.01 covered by 3 papers on ICT in physiology.

As shown in Figure 1B and Multimedia Appendix 5, universities and affiliated hospitals are the primary institutions that have published ICT in physiology–related papers. Southern Illinois University Edwardsville and Duke University in the United States have published the most papers in this field since 2018 and have significantly contributed to ICT in physiology research. Other institutions, including the University of Washington, University of Texas, Vanderbilt University, Central South University, University of Rochester, University of Pennsylvania, Yale University, University of Washington, and Baylor College of Medicine, contributed 3 research papers each. As seen from the year color bar on the left bottom corner of Figure 1B, most nodes are labeled in orange, indicating that most institutions published these articles in 2021 and 2022. Specifically, most of the studies performed in the United States are labeled in green and yellow, corresponding to earlier years, indicating the pioneering role of universities in the United States for ICT in physiology research; in particular, authors from Southern Illinois University published an ICT in physiology paper in 2017, which is earlier than most institutions contributing to this field.

### Cocitation Analysis of References

The highly cited literature on ICT in physiology is summarized in Table 1, which shows the top 15 most influential articles in this field of research ranked by citation frequency and mediation centrality published between 2013 and 2020. The top-ranked item by citation counts is by Chen et al [21], which was published in 2017 with a citation count of 8 and a centrality of 0.26, followed by the paper published by McLaughlin et al [29] in 2014, also with a citation count of 8.

**Table 1.** Top 12 influential papers on inverted teaching in physiology published in the last decade (2013‐2023).

Cited reference	Citation count	Centrality	Publication year
Chen et al [21]	8	0.26	2017
McLaughlin et al [29]	8	0	2014
Tune et al [22]	5	0	2013
Gilboy et al [30]	4	0.12	2015
Pierce and Fox [31]	4	0.15	2012
Betihavas et al [1]	4	0.5	2016
Xiao et al [32]	4	0.17	2018
Hew and Lo [33]	4	0.2	2018
Day [34]	3	0.07	2018
French et al [35]	3	0.04	2018
Blair et al [36]	3	0.06	2020
Freeman et al [37]	3	0.08	2014
Akçayır and Akçayır [38]	3	0.3	2018
Foldnes [39]	3	0.03	2016
Gross et al [40]	3	0.03	2015

### Cocitation Analysis

As shown in Multimedia Appendix 6 and Multimedia Appendix 7, Gopalan C was found to be the most cited author with a count of 5 and a centrality of 0.02.

Multimedia Appendix 8 summarizes the top 10 journals that published ICT in physiology papers. *Advances in Physiology Education* was the first journal to publish ICT in physiology research papers and has maintained the highest frequency of citations from 2013 to 2022 (also see Multimedia Appendix 6). Additionally, journals such as *Computers & Education* and *The Internet and Higher Education* have also provided considerable attention to this topic, implying that modern educational technologies such as information science and the internet play a crucial role in facilitating the inverted classroom mode.

### Research Hot Spots Suggested by Keyword Co-Occurrence Analysis

**Figure 2.**
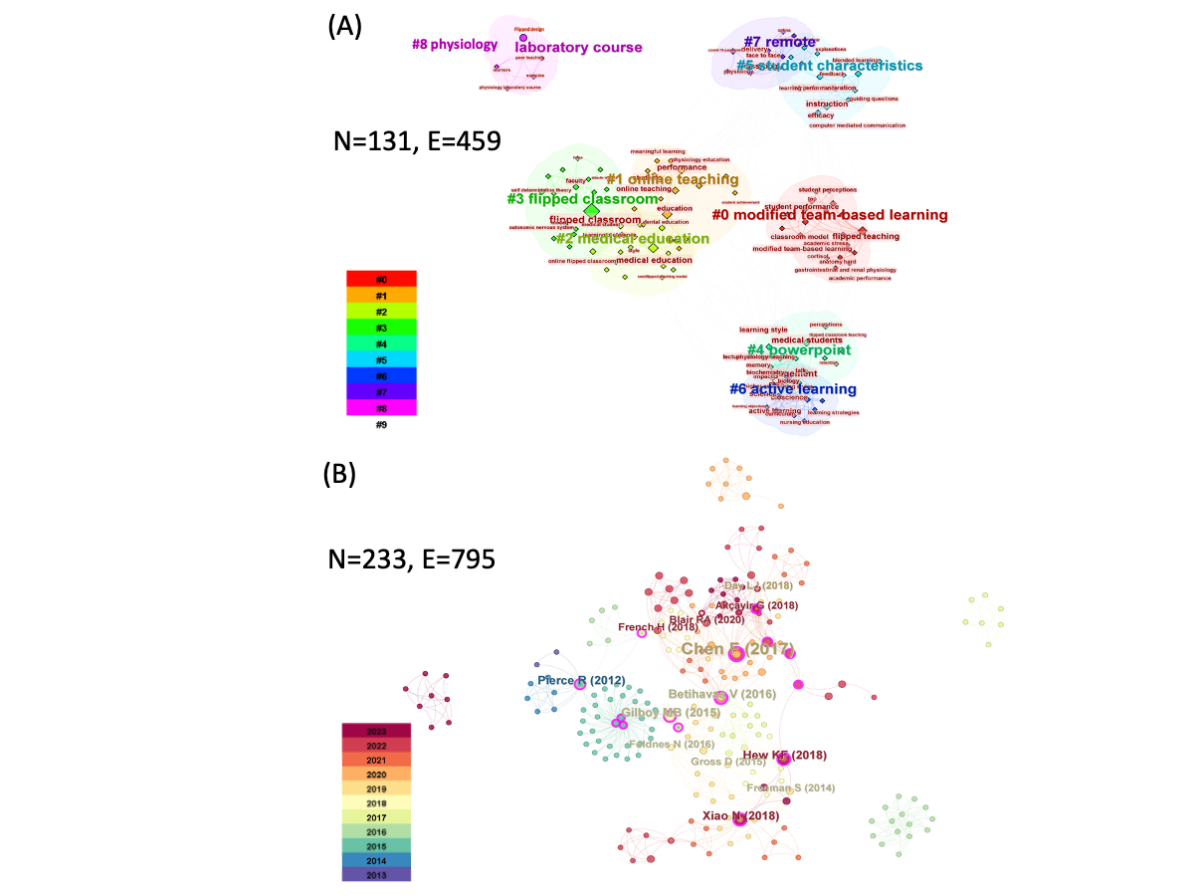
Co-occurrence map and appearance history of keywords in literature related to inverted teaching in physiology. (**A**) The map of keyword clusters and the timeline map (N=131, E=459). (**B**) The co-occurrence map of keywords (N=233, E=795). The node size, N, corresponds to the frequency of publications from each journal. The connecting lines, E, represent collaborative connections between journals, with thicker lines indicating more frequent collaboration.

Figure 2 presents the coexistence diagram of ICT in physiology keywords, with each node representing a keyword and the font size indicating the node’s size; that is, a larger font indicates that the keyword appears more frequently. The cluster labels obtained from the keyword cluster analysis can indirectly reflect the leading research topics, while the timeline map of the keyword clusters can demonstrate the leading research topics by time. Table 2 lists the top keyword clusters in ICT in physiology research according to the number of occurrences and centrality of each keyword, demonstrating that the top keywords are “flipped classroom,” “active learning,” “student performance,” “performance,” and “medical education.” Figure 2A shows that from 2013 to 2022, research on ICT in physiology focused on medical education, performance, engagement, active learning, online teaching, and other aspects. According to the intermediary centrality, “flipped classroom” (0.69) is the most influential keyword, followed by “medical education” (0.2) and “education” (0.14).

**Table 2.** Main keywords in research related to inverted teaching in physiology.

Keywords	Co-occurrence number	Mediator centrality	First year of appearance
flipped classroom	22	0.69	2013
medical education	8	0.2	2016
education	7	0.14	2020
performance	6	0.14	2014
engagement	6	0.26	2015
flipped teaching	5	0.19	2018
student performance	4	0.04	2013
medical students	4	0.19	2016
active learning	4	0.05	2016
online teaching	3	0.06	2021
instruction	3	0.11	2015
classroom	3	0.09	2015
modified team-based learning	2	0.02	2017
dental education	2	0	2017
classroom model	2	0.05	2017
science	2	0.03	2017
faculty	2	0	2021
covid-19 pandemic	2	0	2022
physiology education	2	0.04	2016
bioscience	2	0.01	2019
perceptions	2	0.04	2022
higher education	2	0.01	2019
medical student	2	0.01	2018
students	2	0.01	2015
efficacy	2	0.09	2020
physiology	2	0.02	2015
learning preference	2	0.01	2021
student perceptions	2	0.02	2016
too	2	0	2022
blended learning	2	0.03	2020
online flipped classroom	2	0	2022
intrinsic motivation	2	0.03	2014
self-determination theory	2	0	2021
learning style	2	0.15	2016

The keyword co-occurrence analysis showed that in addition to the retrieved topic term “flipped classroom,” “medical education” ranked the highest in terms of word frequency and ranked the third highest according to mediator centrality, reflecting that active learning is a hot topic in ICT in physiology research. The keywords ”education,” “performance,” and “engagement” followed closely behind, with the centrality being 0.14, 0.14, and 0.26, respectively (Table 2). This indicates that researchers in the field of ICT in physiology have been paying relatively more attention to performance aspects, which could reflect the effectiveness and satisfaction of ICT in physiology. The keywords “engagement” and “perceptions” also had high co-occurrence numbers and mediator centrality.

### Research Hot Spots and Frontier Topics Suggested by Keyword Cluster Analysis

Based on other keywords in the same cluster and the popular words obtained by the latent semantic analysis/indexing algorithm, it was found that many popular words in each cluster reflected the current hot spots of ICT in physiology and had good consistency with the hot spot topics obtained by the co-occurrence analysis of keywords (see Multimedia Appendix 9), such as active learning, self-directed learning, student characteristics, learning preferences, learning styles, modified team-based learning, learning environment, flipped design, student engagement, and undergraduate medical education, among others.

## Discussion

### Principal Findings

In this study, we used CiteSpace software to visually analyze the literature related to the use of ICT in physiology published between 2000 and 2023 retrieved from the WoS database. The results of this bibliometric analysis showed that the core authors publishing in the field of ICT in physiology include staff from universities and affiliated hospitals. Some research teams have also formed cooperative relationships. Research in ICT in physiology mainly focuses on active learning, autonomous learning, student performance, teaching effectiveness, blended teaching, personalized flipped teaching, and other related topics.

Overall, studies on the ICT model in the context of physiology remain scarce, with limited collaboration among authors and a consequent lack of a cohesive research network. Regional growth in this field is uneven and international disparities are evident. Despite the many established benefits of ICT, it is not widely used in various nations and regions. This may be attributable to the fact that the development of the ICT model is still in its infancy, and a mature theoretical structure is needed and must be tested over a wide range of professional specialties. In this sense, relevant researchers must increase interaction and collaboration, investigate systematic teaching techniques appropriate for various disciplines, and perform practical testing and assessment of the model. In the future, research power can be integrated to form a cohesive unit through cooperation among research institutions to promote further breakthroughs in ICT research in the context of physiology.

### Development of ICT in Physiology

The ICT model has undergone three stages of development, including the introduction stage (2013‐2014), extensive practice stage (2015‐2019), and modification and growth stage (2020‐2022).

Several studies have confirmed that an active-learning strategy is associated with improved student performance, a reduced failure rate, and better learning achievements in basic and clinical medical education [37,41]. Shaffer [42] reported that anatomy course objectives were achieved at a much higher rate after incorporating an active teaching style compared to the achievement rate following traditional teaching. Furthermore, in the clinical discipline, Qutub [43] reported the considerable effectiveness of ICT as an active learning style in a hematology course, enabling students to obtain desirable knowledge and improve their academic performance; moreover, students recognized that ICT as an active learning style was more beneficial than the traditional teaching approach. In 2016, Betihavas et al [1] performed a systematic review of 9 studies on the use of ICT in nursing education and reported that nursing students achieved similar or higher academic outcomes with ICT than with a conventional teaching strategy; however, the students indicated a mixed sense of satisfaction.

Other researchers in medical education and health science programs have reported similar results. For example, in an analysis of 274 papers, Barranquero-Herbosa et al [44] found that ICT in nursing education improves performance and is well-received by both students and instructors. O’Connor et al [45] concluded that reversing the flow of classroom teaching improves academic performance, develops self-directed learning skills, and consolidates acquired knowledge through active learning strategies. Sultan [46] found that flipping the classroom gives students more time for active learning, peer collaboration, and applying and analyzing theoretical knowledge. Moreover, McLean et al [47] showed that ICT could improve students’ preparation, attendance, and participation in the course Medical Sciences 4200, an elective nonthesis-based course that covers content related to physiology, biochemistry, and immunology.

With COVID-19 wreaking havoc worldwide in early 2020, the strict and rapid public health measures put forward led to the suspension of face-to-face education and the transfer of the classroom to online meetings, which also corresponds to the application of blended learning as a pedagogical approach based on a combination of online and face-to-face education processes [48]. This necessary shift during the pandemic greatly facilitated the implementation of ICT in various subjects and expanded the use of other types of education tools. For instance, Bawazeer et al [49] reported the use of polls in virtual sessions on physiology, pharmacology, and pathology courses to assess students’ engagement, understanding, performance, and attendance, and found improvements in understanding and permormance. Feng et al [28] reported that incorporating the inverted classroom and a team-based learning strategy in the online setting can enhance the learning outcomes for students in a clinical immunology laboratory course. Although the pandemic and the availability of novel technologies have made blended learning a “new normal” in medical education, the successful adaptation of blended learning requires sufficient teacher training as well as the adoption of appropriate technologies by educational institutions [50].

### The Role of ICT in Medical Education

In 2018, Chung et al [51] performed a systematic review on the use of ICT in nursing education, which showed that the basic flipped classroom mode has been frequently used in nursing education; nevertheless, the effects of ICT on learning behavior in physiology courses were not clearly investigated and only a few studies included in the review reported the use of after-class activities to engage students in facilitating the applications of the knowledge learned. Moreover, Lin and Hwang [52] reviewed studies on ICT papers published up to 2017 based on the technology-enhanced learning model, and noted that little attention was paid to the assessment of learners’ higher-order thinking skills and their degree of preparation or cognitive load. Similar findings have also been reported in relation to the application of ICT in subjects other than medicine, including mathematics [53].

Nevertheless, there is no doubt that ICT can efficiently engage students in learning sessions, even during the pandemic [54]. Research investigating students’ perceptions and performance revealed that students have high levels of acceptance for a virtual flipped teaching approach, which was already evident prior to the COVID-19 pandemic [9,55-57].

### Lack of a Cohesive Research Network in ICT in Physiology Research

Acknowledging the importance of international cooperation and the role of different countries contributing to research on ICT in physiology may facilitate communication and collaboration among countries. With the highest number of published papers, authors from the United States have been the primary contributors to research on the applications of ICT in physiology courses since 2013.

The positive effects of ICT largely depend on an effective classroom design [58]. Designing an effective inverted classroom, guiding students to engage in inverted classroom learning, and personalizing the ICT to enhance teaching effectiveness and student learning outcomes have increasingly become the main topics of ICT research. These are common challenges encountered by teachers and students in ICT. Since a layered teaching approach adapted to the learning, teaching, and classroom conditions can maximize the expected benefits, various ICT approaches have been developed to date, such as partially inverted classrooms [59], Small Private Online Courses–based inverted classrooms [60], and lecture-based inverted classrooms [61].

### Current Hot Spots of ICT in Physiology Research

There are currently three main topics generally discussed in the field of ICT: preparation before class, classroom activities, and consolidation after class [23]. The current hot spots of research in ICT for physiology worldwide focus on active learning, inverted classroom design, student perception and engagement, teaching effectiveness, and teaching evaluation, among others, while the scope of the research includes students, teachers, school teaching management, and national educational guidelines and policies. Moreover, our results are consistent with previous bibliometric studies related to the research on ICT in other fields [62]. For instance, a recent review by Cheng et al [62] on the top 100 highly cited ICT papers similarly showed that researchers in this field have largely focused on students’ learning achievements and learning behaviors rather than directly comparing the benefits of inverted and traditional learning. Similarly, Meral et al [63] reported that motivation, perception, and academic achievement/performance were the most common topics in the ICT studies published between 2010 and 2019.

Regarding the research hot spots suggested by the analysis of keywords, we identified the following main areas of focus of research on ICT in physiology at present: (1) ICT theories, including active learning and independent learning; (2) ICT strategies, including inverted design, student characteristics, learning style, learning preference, learning environment, educational technology, and student participation; and (3) ICT evaluations, including academic performance, student performance, and student satisfaction. Specific to disciplines and programs, the field of research on ICT in physiology covers clinical medicine, stomatology, nursing, pharmacy, and veterinary medicine, among others. With respect to the courses, ICT approaches can be applied to general physiology, gastrointestinal and renal physiology, exercise physiology, physiology lab courses, and introductory biology. The applicable levels of education include graduate, undergraduate, professional training, and adult continuing education.

### Study Strengths and Limitations

This study has both strengths and limitations. To our knowledge, this is the first study to map the current ICT studies in physiology specifically rather than considering the whole field of ICT. Moreover, the visualization of the quantitative results provides a convenient and comprehensible understanding of the current publication status of studies, research hot spots, and development trends in the field of ICT for physiology.

Although all attempts were made to include relevant nouns and terms in the literature retrieval process, some relevant papers may have nevertheless been missed. Additionally, the search only incorporated “physiology” as the keyword for the teaching subject, which may have led to evidence selection bias in which research that covers all medical courses rather than physiology alone may have been missed and could not be incorporated into the study for analysis. In addition, the search was limited to the WoS database, which may have excluded some important non-English publications. Moreover, each subject has unique characteristics in the application of an inverted teaching model, and the results and conclusions reached based on the analysis of this study may not necessarily be generalized to other subjects; thus, these results should be interpreted with caution.

### Conclusion

This study analyzes literature on ICT in physiology, identifying core authors, research topics, and development stages. To date, research in this field has focused on active and autonomous learning, student performance, the teaching effect, blended teaching, and personalized flipping teaching models. The development of ICT is linked to modern information technology, the COVID-19 pandemic, educational teaching concepts, and related teaching reform policies. Based on these findings, further academic exchanges and cooperation in applications of ICT in physiology are encouraged, which can highlight the potential of this teaching model to train the next generation of excellent medical talents.

## Supplementary material

10.2196/52224 Multimedia Appendix 1 Flowchart of literature selection.

10.2196/52224 Multimedia Appendix 2 Excluded studies with reasons.

10.2196/52224 Multimedia Appendix 3 The top 12 authors who published relevant papers on inverted teaching in physiology.

10.2196/52224 Multimedia Appendix 4 Distribution of countries publishing papers related to inverted teaching in physiology.

10.2196/52224 Multimedia Appendix 5 The top 12 institutions publishing papers related to inverted teaching in physiology.

10.2196/52224 Multimedia Appendix 6 (A) The cited reference analysis map of inverted teaching in physiology: N=235, E=684. Node size (N) corresponds to the frequency of publications from each reference. The connecting lines (E) represent collaborative connections between authors, with thicker lines indicating more frequent collaboration. (B) Analysis of cocited journals (N=236, E=996). Node size (N) corresponds to the frequency of publications from each journal. The connecting lines (E) represent citation connections between references, with thicker lines indicating more frequent cocitations.

10.2196/52224 Multimedia Appendix 7 The most influential authors of inverted teaching in physiology research.

10.2196/52224 Multimedia Appendix 8 Primary journals that publish research papers in the field of inverted classroom teaching in physiology.

10.2196/52224 Multimedia Appendix 9 Information of the main clusters of keywords in research related to inverted teaching in physiology.

## Abbreviations

ICT: inverted classroom teaching
WoS: Web of Science

## References

[1] Betihavas V, Bridgman H, Kornhaber R, Cross M (2016). The evidence for ‘flipping out’: a systematic review of the flipped classroom in nursing education. Nurse Educ Today.

[2] Lage MJ, Platt GJ, Treglia M (2000). Inverting the classroom: a gateway to creating an inclusive learning environment. J Econ Educ.

[3] Persky AM, McLaughlin JE (2017). The flipped classroom - from theory to practice in health professional education. Am J Pharm Educ.

[4] Goodman BE, Martin DS, Williams JL (2002). Teaching human cardiovascular and respiratory physiology with the station method. Adv Physiol Educ.

[5] Taylor DCM, Hamdy H (2013). Adult learning theories: implications for learning and teaching in medical education: AMEE guide no. 83. Med Teach.

[6] Ramnanan CJ, Pound LD (2017). Advances in medical education and practice: student perceptions of the flipped classroom. Adv Med Educ Pract.

[7] del Arco I, Mercadé-Melé P, Ramos-Pla A, Flores-Alarcia Ò (2022). Bibliometric analysis of the flipped classroom pedagogical model: trends and strategic lines of study. Front Educ.

[8] Julia J, Afrianti N, Soomro KA (2020). Flipped classroom educational model (2010-2019): a bibliometric study. Eur J Ed Res.

[9] Beason-Abmayr B, Caprette DR, Gopalan C (2021). Flipped teaching eased the transition from face-to-face teaching to online instruction during the COVID-19 pandemic. Adv Physiol Educ.

[10] Pence PL, Franzen SR, Kim MJ (2021). Flipping to motivate: perceptions among prelicensure nursing students. Nurse Educ.

[11] Birgili B, Demir Ö (2022). An explanatory sequential mixed-method research on the full-scale implementation of flipped learning in the first years of the world’s first fully flipped university: departmental differences. Comput Educ.

[12] Foster G, Stagl S (2018). Design, implementation, and evaluation of an inverted (flipped) classroom model economics for sustainable education course. J Clean Prod.

[13] Critz CM, Knight D (2013). Using the flipped classroom in graduate nursing education. Nurse Educ.

[14] Slominski T, Grindberg S, Momsen J (2019). Physiology is hard: a replication study of students' perceived learning difficulties. Adv Physiol Educ.

[15] Colthorpe KL, Abe H, Ainscough L (2018). How do students deal with difficult physiological knowledge?. Adv Physiol Educ.

[16] Wong PC, Chen C, Gorg C, Shneiderman B, Stasko J, Thomas J (2011). Graph analytics-lessons learned and challenges ahead. IEEE Comput Grap Appl.

[17] Journal citation reports. Clarivate.

[18] Rojas-Sánchez MA, Palos-Sánchez PR, Folgado-Fernández JA (2023). Systematic literature review and bibliometric analysis on virtual reality and education. Educ Inf Technol.

[19] Chen C (2014). The CiteSpace Manual.

[20] Chen C (2006). CiteSpace II: detecting and visualizing emerging trends and transient patterns in scientific literature. J Am Soc Inf Sci.

[21] Chen F, Lui AM, Martinelli SM (2017). A systematic review of the effectiveness of flipped classrooms in medical education. Med Educ.

[22] Tune JD, Sturek M, Basile DP (2013). Flipped classroom model improves graduate student performance in cardiovascular, respiratory, and renal physiology. Adv Physiol Educ.

[23] Sun L, Yang L, Wang X, Zhu J, Zhang X (2022). Hot topics and frontier evolution in college flipped classrooms based on mapping knowledge domains. Front Public Health.

[24] Bingen HM, Steindal SA, Krumsvik R, Tveit B (2019). Nursing students studying physiology within a flipped classroom, self-regulation and off-campus activities. Nurse Educ Pract.

[25] Bingen HM, Tveit B, Krumsvik RJ, Steindal SA (2019). Nursing students’ experiences with the use of a student response system when learning physiology. Nordic J Digit Literacy.

[26] Bingen HM, Steindal SA, Krumsvik RJ, Tveit B (2020). Studying physiology within a flipped classroom: the importance of on-campus activities for nursing students' experiences of mastery. J Clin Nurs.

[27] Xu Y, Chen C, Feng D, Luo Z (2022). A survey of college students on the preference for online teaching videos of variable durations in online flipped classroom. Front Public Health.

[28] Feng Y, Zhao B, Zheng J, Fu Y, Jiang Y (2022). Online flipped classroom with team-based learning promoted learning activity in a clinical laboratory immunology class: response to the COVID-19 pandemic. BMC Med Educ.

[29] McLaughlin JE, Roth MT, Glatt DM (2014). The flipped classroom: a course redesign to foster learning and engagement in a health professions school. Acad Med.

[30] Gilboy MB, Heinerichs S, Pazzaglia G (2015). Enhancing student engagement using the flipped classroom. J Nutr Educ Behav.

[31] Pierce R, Fox J (2012). Vodcasts and active-learning exercises in a "flipped classroom" model of a renal pharmacotherapy module. Am J Pharm Educ.

[32] Xiao N, Thor D, Zheng M, Baek J, Kim G (2018). Flipped classroom narrows the performance gap between low- and high-performing dental students in physiology. Adv Physiol Educ.

[33] Hew KF, Lo CK (2018). Flipped classroom improves student learning in health professions education: a meta-analysis. BMC Med Educ.

[34] Day LJ (2018). A gross anatomy flipped classroom effects performance, retention, and higher‐level thinking in lower performing students. Anat Sci Educ.

[35] French H, Gray M, Gillam-Krakauer M (2018). Flipping the classroom: a national pilot curriculum for physiology in neonatal–perinatal medicine. J Perinatol.

[36] Blair RA, Caton JB, Hamnvik OP (2020). A flipped classroom in graduate medical education. Clin Teach.

[37] Freeman S, Eddy SL, McDonough M (2014). Active learning increases student performance in science, engineering, and mathematics. Proc Natl Acad Sci U S A.

[38] Akçayır G, Akçayır M (2018). The flipped classroom: a review of its advantages and challenges. Comput Educ.

[39] Foldnes N (2016). The flipped classroom and cooperative learning: evidence from a randomised experiment. Act Learn High Educ.

[40] Gross D, Pietri ES, Anderson G, Moyano-Camihort K, Graham MJ (2015). Increased preclass preparation underlies student outcome improvement in the flipped classroom. CBE Life Sci Educ.

[41] Kyriakoulis K, Patelarou A, Laliotis A (2016). Educational strategies for teaching evidence-based practice to undergraduate health students: systematic review. J Educ Eval Health Prof.

[42] Shaffer JF (2016). Student performance in and perceptions of a high structure undergraduate human anatomy course. Anat Sci Educ.

[43] Qutob H (2022). Effect of flipped classroom approach in the teaching of a hematology course. PLoS One.

[44] Barranquero-Herbosa M, Abajas-Bustillo R, Ortego-Maté C (2022). Effectiveness of flipped classroom in nursing education: a systematic review of systematic and integrative reviews. Int J Nurs Stud.

[45] O’Connor EE, Fried J, McNulty N (2016). Flipping radiology education right side up. Acad Radiol.

[46] Sultan AS (2018). The flipped classroom: an active teaching and learning strategy for making the sessions more interactive and challenging. J Pak Med Assoc.

[47] McLean S, Attardi SM, Faden L, Goldszmidt M (2016). Flipped classrooms and student learning: not just surface gains. Adv Physiol Educ.

[48] Tonbuloğlu B, Tonbuloğlu İ (2023). Trends and patterns in blended learning research (1965-2022). Educ Inf Technol.

[49] Bawazeer MA, Aamir S, Othman F, Alkahtani R (2023). Students engagement using polls in virtual sessions of physiology, pathology, and pharmacology at King Saud bin Abdulaziz University for Health Sciences during COVID-19 pandemic: a cross-sectional study. BMC Med Educ.

[50] Bozkurt A (2022). A retro perspective on blended/hybrid learning: systematic review, mapping and visualization of the scholarly landscape. J Interact Media Educ.

[51] Chung CJ, Lai CL, Hwang GJ (2021). Roles and research trends of flipped classrooms in nursing education: a review of academic publications from 2010 to 2017. Interact Learn Environ.

[52] Lin HC, Hwang GJ (2019). Research trends of flipped classroom studies for medical courses: a review of journal publications from 2008 to 2017 based on the technology-enhanced learning model. Interact Learn Environ.

[53] Yang QF, Lin CJ, Hwang GJ (2021). Research focuses and findings of flipping mathematics classes: a review of journal publications based on the technology-enhanced learning model. Interact Learn Environ.

[54] Gopalan C, Daughrity S, Hackmann E (2022). The past, the present, and the future of flipped teaching. Adv Physiol Educ.

[55] Gopalan C, Butts-Wilmsmeyer C, Moran V (2021). Virtual flipped teaching during the COVID-19 pandemic. Adv Physiol Educ.

[56] Bryson JR, Andres L (2020). Covid-19 and rapid adoption and improvisation of online teaching: curating resources for extensive versus intensive online learning experiences. J Geogr High Educ.

[57] Fogg KC, Maki SJ (2021). A remote flipped classroom approach to teaching introductory biomedical engineering during COVID-19. Biomed Eng Educ.

[58] Paralikar S, Shah CJ, Joshi A, Kathrotia R (2022). Acquisition of higher-order cognitive skills (HOCS) using the flipped classroom model: a quasi-experimental study. Cureus.

[59] Lax N, Morris J, Kolber BJ (2017). A partial flip classroom exercise in a large introductory general biology course increases performance at multiple levels. J Biol Educ.

[60] Zhang XM, Yu JY, Yang Y, Feng CP, Lyu J, Xu SL (2019). A flipped classroom method based on a small private online course in physiology. Adv Physiol Educ.

[61] Ulrich LM, Palacios S, Kirkby SE (2022). Flipped classroom vs. engaging lecture style for pulmonary physiology. Am J Respir Crit Care Med.

[62] Cheng SC, Hwang GJ, Lai CL (2022). Critical research advancements of flipped learning: a review of the top 100 highly cited papers. Interact Learn Environ.

[63] Meral E, Teke D, Güler M, Namli ZB (2020). General trends of studies on flipped classroom model: bibliometric mapping and content analysis. Int Online J Educ Teach.

