# Supplementary figures and images for: Inverted Classroom Teaching of Physiology in Basic Medical Education: Bibliometric Visual Analysis
Source: JMIR Med Educ. 2024 Jun 25;10:e52224. doi: 10.2196/52224 (PMC11217164; doi:10.2196/52224)

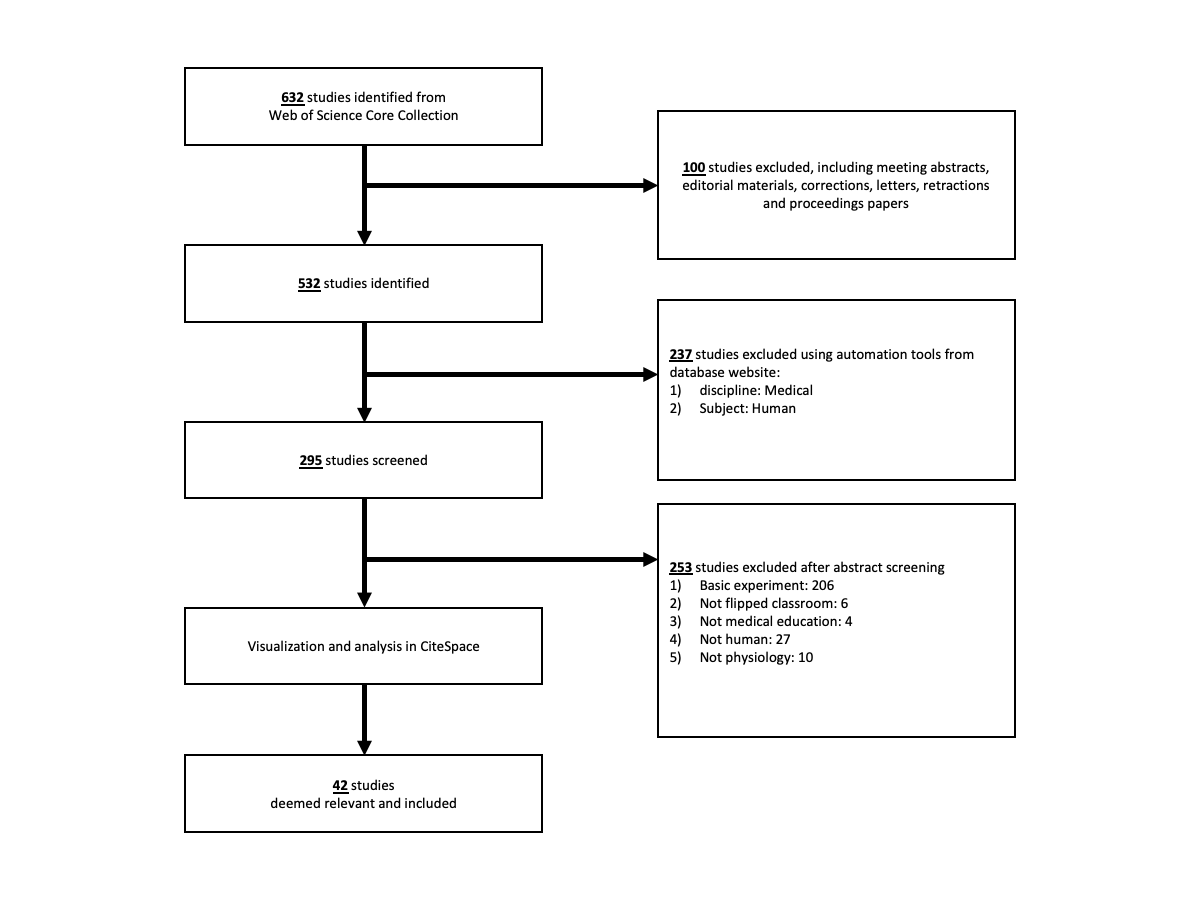

Supplement: Multimedia Appendix 1 [file mededu-v10-e52224-s001.png]

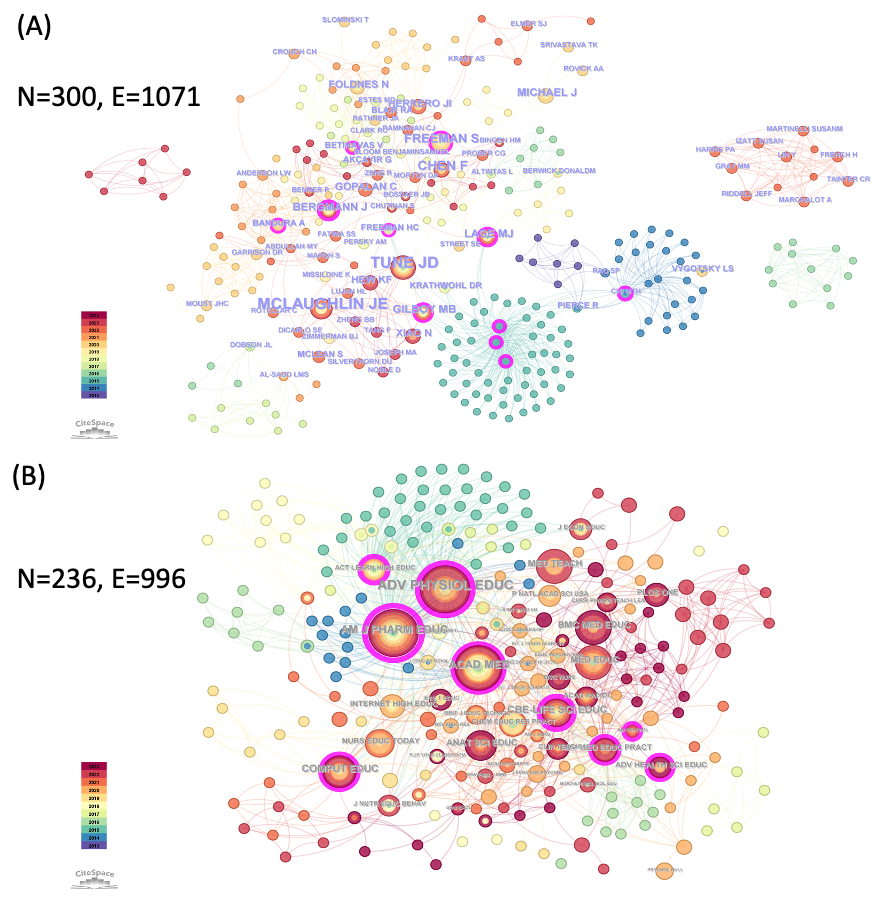

Supplement: Multimedia Appendix 6 [file mededu-v10-e52224-s006.png]
