# Supplementary material for: Inverted Classroom Teaching of Physiology in Basic Medical Education: Bibliometric Visual Analysis
Source: JMIR Med Educ. 2024 Jun 25;10:e52224. doi: 10.2196/52224 (PMC11217164; doi:10.2196/52224)
Supplement: Multimedia Appendix 2 [file mededu-v10-e52224-s002.docx]

**Excluded studies with reasons.**

| **No.** | **Author** | **Year** | **Title** | **Journal** | **DOI** | **Reasons for exclusion** |
| --- | --- | --- | --- | --- | --- | --- |
| 1 | Albarran, L., J. J. Lopez, I. Jardin, et al. | 2018 | Efhb Is a Novel Cytosolic Ca<Sup>2+</Sup> Sensor That Modulates Stim1-Saraf Interaction | Cellular Physiology and Biochemistry | 10.1159/000495494 | basic experiment |
| 2 | Alexander, S. and M. Hannink | 2019 | Exploring the Molecular Genetic Foundations of Cancer Biology and How Biomedical Advances Are Made in an Advanced Undergraduate Course | Biochemistry and Molecular Biology Education | 10.1002/bmb.21247 | basic experiment |
| 3 | Alvin, Z., G. G. Laurence, B. R. Coleman, et al. | 2011 | Regulation of L-Type Inward Calcium Channel Activity by Captopril and Angiotensin Ii Via the Phosphatidyl Inositol 3-Kinase Pathway in Cardiomyocytes from Volume-Overload Hypertrophied Rat Hearts | Canadian Journal of Physiology and Pharmacology | 10.1139/y11-011 | basic experiment |
| 4 | Andjelic, S., G. Zupancic, D. Perovsek, et al. | 2010 | Anterior Lens Capsule as a Tool to Study the Physiology of Human Lens Epithelial Cells | Zdravniski Vestnik-Slovenian Medical Journal / | | basic experiment |
| 5 | Atack, J. M., C. Y. Guo, T. Litfin, et al. | 2020 | Systematic Analysis of Rebase Identifies Numerous Type I Restriction-Modification Systems with Duplicated, Distinct <I>Hsds</I> Specificity Genes That Can Switch System Specificity by Recombination | Msystems | 10.1128/mSystems.00497-20 | basic experiment |
| 6 | Bai, L., Z. B. Yu, C. Z. Wang, et al. | 2011 | Dual Role of Tgf-Β1 on Fas-Induced Apoptosis in Lung Epithelial Cells | Respiratory Physiology & Neurobiology | 10.1016/j.resp.2011.04.016 | basic experiment |
| 7 | Beyer, M., S. Vandersee, I. Cosagarea, et al. | 2016 | The Effects of Arsenic Trioxide in Combination with Retinoic Acids on Cutaneous T-Cell Lymphoma Cell Lines | Skin Pharmacology and Physiology | 10.1159/000443840 | basic experiment |
| 8 | Bharhani, M. S., R. Borojevic, S. Basak, et al. | 2006 | Il-10 Protects Mouse Intestinal Epithelial Cells from Fas-Induced Apoptosis Via Modulating Fas Expression and Altering Caspase-8 and Flip Expression | American Journal of Physiology-Gastrointestinal and Liver Physiology | 10.1152/ajpgi.00438.2005 | basic experiment |
| 9 | Blair, C., M. Sulik, M. Willoughby, et al. | 2015 | Catechol-<I>O</I>-Methyltransferase Val158met Polymorphism Interacts with Early Experience to Predict Executive Functions in Early Childhood | Developmental Psychobiology | 10.1002/dev.21332 | basic experiment |
| 10 | Bogle, J. M., D. Barrs, M. Wester, et al. | 2016 | Labyrinthine Concussion Following Gunshot Injury: A Case Report | International Journal of Audiology | 10.3109/14992027.2016.1166398 | basic experiment |
| 11 | Bolzoni, F., R. Esposti, S. M. Marchese, et al. | 2018 | Disrupt of Intra-Limb Apa Pattern in Parkinsonian Patients Performing Index-Finger Flexion | Frontiers in Physiology | 10.3389/fphys.2018.01745 | basic experiment |
| 12 | Boondam, Y., P. Songvut, M. H. Tantisira, et al. | 2019 | Inverted U-Shaped Response of a Standardized Extract of <I>Centella Asiatica</I> (Eca 233) on Memory Enhancement | Scientific Reports | 10.1038/s41598-019-44867-z | basic experiment |
| 13 | Booth, L., J. L. Roberts, N. Cruickshanks, et al. | 2015 | Pde5 Inhibitors Enhance Celecoxib Killing in Multiple Tumor Types | Journal of Cellular Physiology | 10.1002/jcp.24843 | basic experiment |
| 14 | Borracci, R. A., J. D. M. Pulvet, C. A. Ingino, et al. | 2018 | Geometric Patterns of Time-Delay Plots from Different Cardiac Rhythms and Arrhythmias Using Short-Term Ekg Signals | Clinical Physiology and Functional Imaging | 10.1111/cpf.12494 | basic experiment |
| 15 | Cao, Y., S. Adhikari, A. D. Ang, et al. | 2006 | Mechanism of Induction of Pancreatic Acinar Cell Apoptosis by Hydrogen Sulfide | American Journal of Physiology-Cell Physiology | 10.1152/ajpcell.00547.2005 | basic experiment |
| 16 | Carlisle, R. E. and A. D. Kuo | 2023 | Optimization of Energy and Time Predicts Dynamic Speeds for Human Walking | Elife | 10.7554/eLife.81939 | basic experiment |
| 17 | Carlson, D. A., P. J. Kahrilas, K. Ritter, et al. | 2018 | Mechanisms of Repetitive Retrograde Contractions in Response to Sustained Esophageal Distension: A Study Evaluating Patients with Postfundoplication Dysphagia | American Journal of Physiology-Gastrointestinal and Liver Physiology | 10.1152/ajpgi.00368.2017 | basic experiment |
| 18 | Carlson, D. A., W. J. Kou, M. Masihi, et al. | 2020 | Repetitive Antegrade Contraction: A Novel Response to Sustained Esophageal Distension Is Modulated by Cholinergic Influence | American Journal of Physiology-Gastrointestinal and Liver Physiology | 10.1152/ajpgi.00305.2020 | basic experiment |
| 19 | Catanzaro, J. N., P. M. Meraj, S. Y. Zheng, et al. | 2008 | Electrocardiographic T-Wave Changes Underlying Acute Cardiac and Cerebral Events | American Journal of Emergency Medicine | 10.1016/j.ajem.2007.10.017 | basic experiment |
| 20 | Chanvorachote, P. and V. Pongrakhananon | 2013 | Ouabain Downregulates Mcl-1 and Sensitizes Lung Cancer Cells to Trail-Induced Apoptosis | American Journal of Physiology-Cell Physiology | 10.1152/ajpcell.00225.2012 | basic experiment |
| 21 | Chen, J., C. L. Wu, B. L. Zhang, et al. | 2018 | <I>Piggybac</I> Transposon-Mediated Transgenesis in the Pacific Oyster (<I>Crassostrea Gigas</I>) - First Time in Mollusks | Frontiers in Physiology | 10.3389/fphys.2018.00811 | basic experiment |
| 22 | Chen, P., W. L. Gu, M. Z. Gong, et al. | 2017 | Artesunate Decreases Β-Catenin Expression, Cell Proliferation and Apoptosis Resistance in the Mg-63 Human Osteosarcoma Cell Line | Cellular Physiology and Biochemistry | 10.1159/000484118 | basic experiment |
| 23 | Clarke, A. L., S. Petrou, J. V. Walsh, et al. | 2003 | Site of Action of Fatty Acids and Other Charged Lipids on Bk<Sub>Ca</Sub> Channels from Arterial Smooth Muscle Cells | American Journal of Physiology-Cell Physiology | 10.1152/ajpcell.00364.2002 | basic experiment |
| 24 | Cronemberger, S., A. C. L. da Silva and N. Calixto | 2010 | Importance of Intraocular Pressure Measurement at 6:00 A.M. In Bed and in Darkness in Suspected and Glaucomatous Patients | Arquivos Brasileiros De Oftalmologia | 10.1590/s0004-27492010000400009 | basic experiment |
| 25 | Cronin, J. N., D. C. Crockett, G. Perchiazzi, et al. | 2023 | Intra-Tidal Pao<Sub>2</Sub> Oscillations Associated with Mechanical Ventilation: A Pilot Study to Identify Discrete Morphologies in a Porcine Model | Intensive Care Medicine Experimental | 10.1186/s40635-023-00544-0 | basic experiment |
| 26 | Crow, T., J. Ta, S. Nojoomi, et al. | 2020 | Gene Regulatory Effects of a Large Chromosomal Inversion in Highland Maize | Plos Genetics | 10.1371/journal.pgen.1009213 | basic experiment |
| 27 | Dallmann, R. and N. Mrosovsky | 2006 | Scheduled Wheel Access During Daytime: A Method for Studying Conflicting Zeitgebers | Physiology & Behavior | 10.1016/j.physbeh.2006.04.022 | basic experiment |
| 28 | Dargelos, E., V. Renaud, M. Decossas, et al. | 2018 | Caveolae-Mediated Effects of Tnf-Α on Human Skeletal Muscle Cells | Experimental Cell Research | 10.1016/j.yexcr.2018.07.027 | basic experiment |
| 29 | Dasgupta, T., D. H. Croll, J. A. Owen, et al. | 2014 | A Fundamental Trade-Off in Covalent Switching and Its Circumvention by Enzyme Bifunctionality in Glucose Homeostasis | Journal of Biological Chemistry | 10.1074/jbc.M113.546515 | basic experiment |
| 30 | Dave, M., J. Levin, S. W. Ruffins, et al. | 2022 | A Novel Egg-in-Cube System Enables Long-Term Culture and Dynamic Imaging of Early Embryonic Development | Frontiers in Physiology | 10.3389/fphys.2022.893736 | basic experiment |
| 31 | Dawson, P. A. and D. Markovich | 2002 | Regulation of the Mouse <I>Nas1</I> Promoter by Vitamin D and Thyroid Hormone | Pflugers Archiv-European Journal of Physiology | 10.1007/s00424-002-0789-x | basic experiment |
| 32 | de Abreu, R. M., A. M. Catai, B. Cairo, et al. | 2020 | A Transfer Entropy Approach for the Assessment of the Impact of Inspiratory Muscle Training on the Cardiorespiratory Coupling of Amateur Cyclists | Frontiers in Physiology | 10.3389/fphys.2020.00134 | basic experiment |
| 33 | Desir, S., P. Wong, T. Turbyville, et al. | 2019 | Intercellular Transfer of Oncogenic <I>Kras</I> Via Tunneling Nanotubes Introduces Intracellular Mutational Heterogeneity in Colon Cancer Cells | Cancers | 10.3390/cancers11070892 | basic experiment |
| 34 | Dopico, X. C., M. Evangelou, R. C. Ferreira, et al. | 2015 | Widespread Seasonal Gene Expression Reveals Annual Differences in Human Immunity and Physiology | Nature Communications | 10.1038/ncomms8000 | basic experiment |
| 35 | Eagle, K. | 2014 | Adhd Impacted by Sulfotransferase (Sult1a) Inhibition from Artificial Food Colors and Plant-Based Foods | Physiology & Behavior | 10.1016/j.physbeh.2014.06.005 | basic experiment |
| 36 | Eleazer, C. D. and R. S. Kelso | 2018 | Influence of Study Approaches and Course Design on Academic Success in the Undergraduate Anatomy Laboratory | Anatomical Sciences Education | 10.1002/ase.1766 | basic experiment |
| 37 | Elisha, G., S. Halder, D. A. Carlson, et al. | 2023 | A Mechanics-Based Perspective on the Pressure-Cross-Sectional Area Loop within the Esophageal Body | Frontiers in Physiology | 10.3389/fphys.2022.1066351 | basic experiment |
| 38 | Estienne, V., N. Brisbarre, S. Blanchin, et al. | 2004 | An in Vitro Model Based on Cell Monolayers Grown on the Underside of Large-Pore Filters in Bicameral Chambers for Studying Thyrocyte-Lymphocyte Interactions | American Journal of Physiology-Cell Physiology | 10.1152/ajpcell.00024.2004 | basic experiment |
| 39 | Fabian, D., A. Bukovská, S. Juhás, et al. | 2009 | Apoptotic Processes and DNA Cytosine Methylation in Mouse Embryos Arrested at the 2-Cell Stage | Zygote | 10.1017/s0967199409005413 | basic experiment |
| 40 | Fassini, A., A. A. Scopinho, L. B. M. Resstel, et al. | 2015 | Κ-Opioid Receptors in the Infralimbic Cortex Modulate the Cardiovascular Responses to Acute Stress | Experimental Physiology | 10.1113/expphysiol.2014.084020 | basic experiment |
| 41 | Feoktistova, M., P. Geserick, D. Panayotova-Dimitrova, et al. | 2012 | Pick Your Poison the Ripoptosome, a Cell Death Platform Regulating Apoptosis and Necroptosis | Cell Cycle | 10.4161/cc.11.3.19060 | basic experiment |
| 42 | Fernandez, R., M. Oliveira-Souza and G. Malnic | 2000 | Na<Sup>+</Sup>-Independent Proton Secretion in Mdck-C11 Cells | Pflugers Archiv-European Journal of Physiology | 10.1007/s004240000411 | basic experiment |
| 43 | Ficici, E., J. D. Faraldo-Gómez, M. L. Jennings, et al. | 2017 | Asymmetry of Inverted-Topology Repeats in the Ae1 Anion Exchanger Suggests an Elevator-Like Mechanism | Journal of General Physiology | 10.1085/jgp.201711836 | basic experiment |
| 44 | Formichi, P., E. Radi, C. Battisti, et al. | 2009 | Apoptosis in Cadasil: An in Vitro Study of Lymphocytes and Fibroblasts from a Cohort of Italian Patients | Journal of Cellular Physiology | 10.1002/jcp.21695 | basic experiment |
| 45 | François-Étienne, S., L. Nicolas, N. Eric, et al. | 2023 | Important Role of Endogenous Microbial Symbionts of Fish Gills in the Challenging but Highly Biodiverse Amazonian Blackwaters | Nature Communications | 10.1038/s41467-023-39461-x | basic experiment |
| 46 | Fynne, L., F. Luft, H. Gregersen, et al. | 2013 | Distensibility of the Anal Canal in Patients with Systemic Sclerosis: A Study with the Functional Lumen Imaging Probe | Colorectal Disease | 10.1111/codi.12063 | basic experiment |
| 47 | Gaitanaki, C., P. Papazafiri and I. Beis | 2003 | The Calpain-Calpastatin System and the Calcium Paradox in the Isolated Perfused Pigeon Heart | Cellular Physiology and Biochemistry | 10.1159/000071868 | basic experiment |
| 48 | Gamo, N. J., M. Wang and A. F. T. Arnsten | 2010 | Methylphenidate and Atomoxetine Enhance Prefrontal Function through Α<Sub>2</Sub>-Adrenergic and Dopamine D<Sub>1</Sub> Receptors | Journal of the American Academy of Child and Adolescent Psychiatry | 10.1016/j.jaac.2010.06.015 | basic experiment |
| 49 | Gao, X. L., J. J. Yang, S. J. Wang, et al. | 2019 | Effects of Rna Interference-Mediated Silencing of Toll-Like Receptor 4 Gene on Proliferation and Apoptosis of Human Breast Cancer Mcf-7 and Mda-Mb-231 Cells: An in Vitro Study | Journal of Cellular Physiology | 10.1002/jcp.26573 | basic experiment |
| 50 | Garbayo, L. S., D. M. Harris, S. M. Fiore, et al. | 2023 | A Metacognitive Confidence Calibration (Mcc) Tool to Help Medical Students Scaffold Diagnostic Reasoning in Decision-Making During High-Fidelity Patient Simulations | Advances in Physiology Education | 10.1152/advan.00156.2021 | basic experiment |
| 51 | Gibbs, W. S., S. M. Garrett, C. C. Beeson, et al. | 2018 | Identification of Dual Mechanisms Mediating 5-Hydroxytryptamine Receptor 1f-Induced Mitochondrial Biogenesis | American Journal of Physiology-Renal Physiology | 10.1152/ajprenal.00324.2017 | basic experiment |
| 52 | Gibellini, D., M. C. Re, C. Ponti, et al. | 2005 | Hiv-1 Tat Protein Concomitantly Down-Regulates Apical Caspase-10 and up-Regulates C-Flip in Lymphoid T Cells: A Potential Molecular Mechanism to Escape Trail Cytotoxicity | Journal of Cellular Physiology | 10.1002/jcp.20252 | basic experiment |
| 53 | Goodwin, S. B., C. B. McCorison, J. R. Cavaletto, et al. | 2016 | The Mitochondrial Genome of the Ethanol-Metabolizing, Wine Cellar Mold <I>Zasmidium</I> <I>Cellare</I> Is the Smallest for a Filamentous Ascomycete | Fungal Biology | 10.1016/j.funbio.2016.05.003 | basic experiment |
| 54 | Gorjan, D., N. Sarabon and J. Babic | 2022 | Inter-Individual Variability in Postural Control During External Center of Mass Stabilization | Frontiers in Physiology | 10.3389/fphys.2021.722732 | basic experiment |
| 55 | Gosak, M., R. Markovic, A. Fajmut, et al. | 2015 | The Analysis of Intracellular and Intercellular Calcium Signaling in Human Anterior Lens Capsule Epithelial Cells with Regard to Different Types and Stages of the Cataract | Plos One | 10.1371/journal.pone.0143781 | basic experiment |
| 56 | Graber, T. G., L. Ferguson-Stegall, J. H. Kim, et al. | 2013 | C57bl/6 Neuromuscular Healthspan Scoring System | Journals of Gerontology Series a-Biological Sciences and Medical Sciences | 10.1093/gerona/glt032 | basic experiment |
| 57 | Guerra, M., M. J. Amorim, C. Brás-Silva, et al. | 2013 | Intraventricular Pressure Gradients Throughout the Cardiac Cycle: Effects of Ischaemia and Modulation by Afterload | Experimental Physiology | 10.1113/expphysiol.2012.066324 | basic experiment |
| 58 | Guo, W., N. Huang, J. Cai, et al. | 2006 | Fatty Acid Transport and Metabolism in Hepg2 Cells | American Journal of Physiology-Gastrointestinal and Liver Physiology | 10.1152/ajpgi.00386.2005 | basic experiment |
| 59 | Hamed, H. A., S. Tavallai, S. Grant, et al. | 2015 | Sorafenib/Regorafenib and Lapatinib Interact to Kill Cns Tumor Cells | Journal of Cellular Physiology | 10.1002/jcp.24689 | basic experiment |
| 60 | Hamed, H. A., Y. Yamaguchi, P. B. Fisher, et al. | 2013 | Sorafenib and Hdac Inhibitors Synergize with Trail to Kill Tumor Cells | Journal of Cellular Physiology | 10.1002/jcp.24362 | basic experiment |
| 61 | Hancock, P. A. | 2022 | In Defense of the Maximal Adaptability Model | Physiology & Behavior | 10.1016/j.physbeh.2022.113844 | basic experiment |
| 62 | Harita, Y., N. Miyauchi, T. Karasawa, et al. | 2006 | Altered Expression of Junctional Adhesion Molecule 4 in Injured Podocytes | American Journal of Physiology-Renal Physiology | 10.1152/ajprenal.00253.2005 | basic experiment |
| 63 | Heese, D., C. Sauvant, H. Holzinger, et al. | 2004 | Apical Expression or Expression in a Non Polarized Cell of Hoat1 Inverses Regulation by Epidermal Growth Factor (Egf) as Compared to Basolateral Hoat1 | Cellular Physiology and Biochemistry | 10.1159/000078109 | basic experiment |
| 64 | Heller, L. J., D. E. Mohrman, J. A. Smith, et al. | 2003 | Multitrack System for Superfusing Isolated Cardiac Myocytes | American Journal of Physiology-Heart and Circulatory Physiology | 10.1152/ajpheart.00914.2002 | basic experiment |
| 65 | Holst, S. C., T. Müller, A. Valomon, et al. | 2017 | Functional Polymorphisms in Dopaminergic Genes Modulate Neurobehavioral and Neurophysiological Consequences of Sleep Deprivation | Scientific Reports | 10.1038/srep45982 | basic experiment |
| 66 | Horstmann, A. | 2017 | It Wasn't Me; It Was My Brain - Obesity-Associated Characteristics of Brain Circuits Governing Decision-Making | Physiology & Behavior | 10.1016/j.physbeh.2017.04.001 | basic experiment |
| 67 | Hu, P., Y. N. Gao, Y. Q. Zhang, et al. | 2023 | Ultrasound Image-Based Deep Learning to Differentiate Tubal-Ovarian Abscess from Ovarian Endometriosis Cyst | Frontiers in Physiology | 10.3389/fphys.2023.1101810 | basic experiment |
| 68 | Huang, J. F., M. Tian, C. J. Lv, et al. | 2011 | Preliminary Studies on Induction of Apoptosis by Abamectin in <I>Spodoptera Frugiperda</I> (Sf9) Cell Line | Pesticide Biochemistry and Physiology | 10.1016/j.pestbp.2011.04.010 | basic experiment |
| 69 | Huang, X. T., J. J. Wang, J. Li, et al. | 2020 | Prevalence of Phase Variable Epigenetic Invertons among Host-Associated Bacteria | Nucleic Acids Research | 10.1093/nar/gkaa907 | basic experiment |
| 70 | Hull, J. J., E. J. Hoffmann, O. P. Perera, et al. | 2012 | Identification of the Western Tarnished Plant Bug (<I>Lygus Hesperus</I>) Olfactory Co-Receptor Orco: Expression Profile and Confirmation of Atypical Membrane Topology | Archives of Insect Biochemistry and Physiology | 10.1002/arch.21042 | basic experiment |
| 71 | Hüttermann, S. and D. Memmert | 2014 | Does the Inverted-U Function Disappear in Expert Athletes? An Analysis of the Attentional Behavior under Physical Exercise of Athletes and Non-Athletes | Physiology & Behavior | 10.1016/j.physbeh.2014.04.020 | basic experiment |
| 72 | Imperiale, A., K. Elbayed, F. M. Moussallieh, et al. | 2013 | Metabolomic Profile of the Adrenal Gland: From Physiology to Pathological Conditions | Endocrine-Related Cancer | 10.1530/erc-13-0232 | basic experiment |
| 73 | Ives, S. J., R. H. I. Andtbacka, R. D. Noyes, et al. | 2011 | Human Skeletal Muscle Feed Arteries Studied <I>in Vitro</I>: The Effect of Temperature on Α<Sub>1</Sub>-Adrenergic Responsiveness | Experimental Physiology | 10.1113/expphysiol.2011.059329 | basic experiment |
| 74 | Jagtap, N., R. Kalapala and D. N. Reddy | 2020 | Assessment of Pyloric Sphincter Physiology Using Functional Luminal Imaging Probe in Healthy Volunteers | Journal of Neurogastroenterology and Motility | 10.5056/jnm19200 | basic experiment |
| 75 | Jedrzejczak, W. W., R. Milner, L. Olszewski, et al. | 2017 | Heightened Visual Attention Does Not Affect Inner Ear Function as Measured by Otoacoustic Emissions | Peerj | 10.7717/peerj.4199 | basic experiment |
| 76 | Kas, M. J. H. and D. M. Edgar | 2000 | Photic Phase Response Curve in <I>Octodon Degus</I>:: Assessment as a Function of Activity Phase Preference | American Journal of Physiology-Regulatory Integrative and Comparative Physiology | 10.1152/ajpregu.2000.278.5.R1385 | basic experiment |
| 77 | Khalyfa, A., V. A. Poroyko, Z. H. Qiao, et al. | 2017 | Exosomes and Metabolic Functionin Mice Exposed to Alternating Dark-Light Cycles Mimicking Night Shift Work Schedules | Frontiers in Physiology | 10.3389/fphys.2017.00882 | basic experiment |
| 78 | Khan, H. A., K. Mehmood and R. Al-Hamdan | 2012 | Persistent Junctional Reciprocating Tachycardia (Pjrt) | Kuwait Medical Journal | | basic experiment |
| 79 | Kim, Y. R., M. D. Savellano, R. Weissleder, et al. | 2002 | Steady-State and Dynamic Contrast Mr Imaging of Human Prostate Cancer Xenograft Tumors: A Comparative Study | Technology in Cancer Research & Treatment | 10.1177/153303460200100609 | basic experiment |
| 80 | Klein, H., L. Garneau, U. Banderali, et al. | 2007 | Structural Determinants of the Closed Kca3.1 Channel Pore in Relation to Channel Gating:: Results from a Substituted Cysteine Accessibility Analysis | Journal of General Physiology | 10.1085/jgp.200609726 | basic experiment |
| 81 | Kolb, J. M., F. Pessorrusso, S. Pisipati, et al. | 2023 | Role of Short Interval Flip Panometry in Predicting Long-Term Outcomes after Per-Oral Endoscopic Myotomy | Surgical Endoscopy and Other Interventional Techniques | 10.1007/s00464-023-10319-z | basic experiment |
| 82 | Kong, Z. Y., X. J. Zhu, S. H. Zhang, et al. | 2012 | Phase Contrast Microscopy of Living Cells within the Whole Lens: Spatial Correlations and Morphological Dynamics | Molecular Vision | | basic experiment |
| 83 | Kongsuphol, P., D. Cassidy, F. Romeiras, et al. | 2010 | Metformin Treatment of Diabetes Mellitus Increases the Risk for Pancreatitis in Patients Bearing the Cftr-Mutation S573c | Cellular Physiology and Biochemistry | 10.1159/000303043 | basic experiment |
| 84 | Kothari, M., U. Somashekhar, R. Kothari, et al. | 2023 | 'Flip-Flap' Hernia Sac Sandwich Mesh Hernioplasty for Giant Incisional Hernia | Tropical Doctor | 10.1177/00494755231156494 | basic experiment |
| 85 | Kotula-Balak, M., A. Milon, P. Pawlicki, et al. | 2018 | Insights into the Role of Estrogen-Related Receptors Α, Β and Γ in Tumor Leydig Cells | Tissue & Cell | 10.1016/j.tice.2018.04.003 | basic experiment |
| 86 | Kroeker, C. A. G., S. Adeeb, N. G. Shrive, et al. | 2006 | Compression Induced by Rv Pressure Overload Decreases Regional Coronary Blood Flow in Anesthetized Dogs | American Journal of Physiology-Heart and Circulatory Physiology | 10.1152/ajpheart.01140.2005 | basic experiment |
| 87 | Kroeker, C. A. G., S. Adeeb, J. V. Tyberg, et al. | 2006 | A 2d Fe Model of the Heart Demonstrates the Role of the Pericardium in Ventricular Deformation | American Journal of Physiology-Heart and Circulatory Physiology | 10.1152/ajpheart.00077.2006 | basic experiment |
| 88 | Kuebler, W. M., M. Mertens and A. R. Pries | 2007 | A Two-Component Simulation Model to Teach Respiratory Mechanics | Advances in Physiology Education | 10.1152/advan.00001.2007 | basic experiment |
| 89 | Kuzmiszyn, A. K., A. L. Selli, N. Smaglyukova, et al. | 2022 | Treatment of Cardiovascular Dysfunction with Pde3-Inhibitors in Moderate and Severe Hypothermia-Effects on Cellular Elimination of Cyclic Adenosine Monophosphate and Cyclic Guanosine Monophosphate | Frontiers in Physiology | 10.3389/fphys.2022.923091 | basic experiment |
| 90 | Kyläniemi, M. K., R. Kaukonen, J. Myllyviita, et al. | 2014 | The Regulation and Role of C-Flip in Human Th Cell Differentiation | Plos One | 10.1371/journal.pone.0102022 | basic experiment |
| 91 | Lee, J. H., J. S. Lee, S. Kim, et al. | 2017 | Comparison of Cytotoxicities and Wound Healing Effects of Diquafosol Tetrasodium and Hyaluronic Acid on Human Corneal Epithelial Cells | Korean Journal of Physiology & Pharmacology | 10.4196/kjpp.2017.21.2.189 | basic experiment |
| 92 | Lee, P. H., J. K. Song, B. J. Sun, et al. | 2010 | Outcomes of Patients with Stress-Induced Cardiomyopathy Diagnosed by Echocardiography in a Tertiary Referral Hospital | Journal of the American Society of Echocardiography | 10.1016/j.echo.2010.05.002 | basic experiment |
| 93 | Lee, S., M. Mele, P. Vahl, et al. | 2015 | Na<Sup>+</Sup>,Hco<Sub>3</Sub> <Sup>-</Sup>-Cotransport Is Functionally Upregulated During Human Breast Carcinogenesis and Required for the Inverted Ph Gradient across the Plasma Membrane | Pflugers Archiv-European Journal of Physiology | 10.1007/s00424-014-1524-0 | basic experiment |
| 94 | Leo, F., T. Suvorava, S. K. Heuser, et al. | 2021 | Red Blood Cell and Endothelial Enos Independently Regulate Circulating Nitric Oxide Metabolites and Blood Pressure | Circulation | 10.1161/circulationaha.120.049606 | basic experiment |
| 95 | Lewinson, O., E. Padant and E. Bibi | 2004 | Alkalitolerance: A Biological Function for a Multidrug Transporter in Ph Homeostasis | Proceedings of the National Academy of Sciences of the United States of America | 10.1073/pnas.0405375101 | basic experiment |
| 96 | Li, C., H. Y. Zhang, Y. Liang, et al. | 2018 | Effects of Levonorgestrel and Progesterone on Oviductal Physiology in Mammals | Reproductive Biology and Endocrinology | 10.1186/s12958-018-0377-3 | basic experiment |
| 97 | Li, L. F., Y. F. Li, Z. Y. Dai, et al. | 2016 | Lipid Metabolism in Vascular Smooth Muscle Cells Influenced by Hcmv Infection | Cellular Physiology and Biochemistry | 10.1159/000447880 | basic experiment |
| 98 | Li, X. B., M. W. Wu, D. W. An, et al. | 2019 | Suppression of Tafazzin Promotes Thyroid Cancer Apoptosis Via Activating the Jnk Signaling Pathway and Enhancing Inf2-Mediated Mitochondrial Fission | Journal of Cellular Physiology | 10.1002/jcp.28287 | basic experiment |
| 99 | Li, Y. X., X. M. Su, X. B. Wang, et al. | 2014 | Cytotoxic Effect of Protoporphyrin Ix to Human Leukemia U937 Cells under Ultrasonic Irradiation | Cellular Physiology and Biochemistry | 10.1159/000358687 | basic experiment |
| 100 | Liu, W., Q. Meng, Y. T. Sun, et al. | 2018 | Targeting P-Glycoprotein: Nelfinavir Reverses Adriamycin Resistance in K562/Adr Cells | Cellular Physiology and Biochemistry | 10.1159/000495650 | basic experiment |
| 101 | Liu, X., Y. Zheng, N. M. Samoshina, et al. | 2012 | Fliposomes: Ph-Triggered Conformational Flip of New <I>Trans</I>-2-Aminocyclohexanol-Based Amphiphiles Causes Instant Cargo Release in Liposomes | Journal of Liposome Research | 10.3109/08982104.2012.698420 | basic experiment |
| 102 | Liu, Y. N., J. Z. Yuan, T. Y. Tan, et al. | 2014 | Genetic Inhibition of Protein Kinase Cε Attenuates Necrosis in Experimental Pancreatitis | American Journal of Physiology-Gastrointestinal and Liver Physiology | 10.1152/ajpgi.00432.2013 | basic experiment |
| 103 | Longpré, J. P., L. J. Sasseville and J. Y. Lapointe | 2012 | Simulated Annealing Reveals the Kinetic Activity of Sglt1, a Member of the Leut Structural Family | Journal of General Physiology | 10.1085/jgp.201210822 | basic experiment |
| 104 | Lorusso, D., H. N. Nikolov, D. W. Holdsworth, et al. | 2020 | Vibration of Osteoblastic Cells Using a Novel Motion-Control Platform Does Not Acutely Alter Cytosolic Calcium, but Desensitizes Subsequent Responses to Extracellular Atp | Journal of Cellular Physiology | 10.1002/jcp.29378 | basic experiment |
| 105 | Lu, J. and M. A. Greco | 2006 | Sleep Circuitry and the Hypnotic Mechanism of Gaba<Sub>a</Sub> Drugs | Journal of Clinical Sleep Medicine | | basic experiment |
| 106 | Lyon, A., R. Ariga, A. Mincholé, et al. | 2018 | Distinct Ecg Phenotypes Identified in Hypertrophic Cardiomyopathy Using Machine Learning Associate with Arrhythmic Risk Markers | Frontiers in Physiology | 10.3389/fphys.2018.00213 | basic experiment |
| 107 | Maass, D. L., J. White, B. Sanders, et al. | 2005 | Role of Cytosolic Vs. Mitochondrial Ca<Sup>2+</Sup> Accumulation in Burn Injury-Related Myocardial Inflammation and Function | American Journal of Physiology-Heart and Circulatory Physiology | 10.1152/ajpheart.00367.2004 | basic experiment |
| 108 | Macedo, R., J. L. Fernandes, S. S. Andrade, et al. | 2013 | Morphological and Functional Measurements of the Heart Obtained by Magnetic Resonance Imaging in Brazilians | Arquivos Brasileiros De Cardiologia | 10.5935/abc.20130113 | basic experiment |
| 109 | Malhotra, R., Z. W. Lin, C. Vincenz, et al. | 2001 | Hypoxia Induces Apoptosis Via Two Independent Pathways in Jurkat Cells: Differential Regulation by Glucose | American Journal of Physiology-Cell Physiology | 10.1152/ajpcell.2001.281.5.C1596 | basic experiment |
| 110 | Mané, M. and M. Müller | 2012 | Temporo-Spectral Imaging of Intrinsic Optical Signals During Hypoxia-Induced Spreading Depression-Like Depolarization | Plos One | 10.1371/journal.pone.0043981 | basic experiment |
| 111 | Marabelli, A., R. Lape and L. Sivilotti | 2015 | Mechanism of Activation of the Prokaryotic Channel Elic by Propylamine: A Single-Channel Study | Journal of General Physiology | 10.1085/jgp.201411234 | basic experiment |
| 112 | Marshall, S. E., S. M. Tweedt, C. H. Greene, et al. | 2000 | An Alternative to Synthetic Aortic Grafts Using Jejunum | Journal of Investigative Surgery / | | basic experiment |
| 113 | McLaughlin, J. E., J. Khanova, A. Persky, et al. | 2017 | Design, Implementation, and Outcomes of a Three-Week Pharmacy Bridging Course | American Journal of Pharmaceutical Education | | basic experiment |
| 114 | McMahon, B. P., J. B. Frokjær, P. Kunwald, et al. | 2007 | The Functional Lumen Imaging Probe (Flip) for Evaluation of the Esophagogastric Junction | American Journal of Physiology-Gastrointestinal and Liver Physiology | 10.1152/ajpgi.00311.2006 | basic experiment |
| 115 | Milyutina, Y. P., A. V. Korenevskii, V. V. Vasilyeva, et al. | 2022 | Caspase Activation in Trophoblast Cells after Interacting with Microparticles Produced by Natural Killer Cells in Vitro | Journal of Evolutionary Biochemistry and Physiology | 10.1134/s002209302206014x | basic experiment |
| 116 | Mohammadzadeh, T., S. M. Sadjjadi and H. Rahimi | 2014 | Still and Moving Image Evidences for Mating of <I>Echinococcus Granulosus</I> Reared in Culture Media | Iranian Journal of Parasitology | | basic experiment |
| 117 | Monteiro, J. P., A. F. Martins, M. Lúcio, et al. | 2011 | Nimesulide Interaction with Membrane Model Systems: Are Membrane Physical Effects Involved in Nimesulide Mitochondrial Toxicity? | Toxicology in Vitro | 10.1016/j.tiv.2011.05.014 | basic experiment |
| 118 | Monteiro, O., A. Bhaskar, A. K. M. Ng, et al. | 2021 | Computer-Based Virtual Laboratory Simulations: Labheart Cardiac Physiology Practical | Advances in Physiology Education | 10.1152/advan.00094.2021 | basic experiment |
| 119 | Moorhouse, A. J., A. Keramidas, A. Zaykin, et al. | 2002 | Single Channel Analysis of Conductance and Rectification in Cation-Selective, Mutant Glycine Receptor Channels | Journal of General Physiology | 10.1085/jgp.20028553 | basic experiment |
| 120 | Morel, O., L. Jesel, J. M. Freyssinet, et al. | 2011 | Cellular Mechanisms Underlying the Formation of Circulating Microparticles | Arteriosclerosis Thrombosis and Vascular Biology | 10.1161/atvbaha.109.200956 | basic experiment |
| 121 | Nakamura, T., T. Horikoshi and K. Kugiyama | 2020 | Relationship of a Thinned Medial Layer to the Attenuated Contractile Response in Atherosclerotic Coronary Arteries | American Journal of Physiology-Heart and Circulatory Physiology | 10.1152/ajpheart.00537.2019 | basic experiment |
| 122 | Nakayama, Y., Y. Nakajima, N. Kato, et al. | 2006 | Insulin-Like Growth Factor-1 Increases Bone Sialoprotein (Bsp) Expression through Fibroblast Growth Factor-2 Response Element and Homeodomain Protein-Binding Site in the Proximal Promoter of the Bsp Gene | Journal of Cellular Physiology | 10.1002/jcp.20664 | basic experiment |
| 123 | Nishizaki, T. | 2018 | Dioleoylphosphoethanolamine Retains Cell Surface Glut4 by Inhibiting Pkcα-Driven Internalization | Cellular Physiology and Biochemistry | 10.1159/000489439 | basic experiment |
| 124 | Ogasawara, K., T. Terada, J. Asaka, et al. | 2007 | Hepatocyte Nuclear Factor-4α Regulates the Human Organic Anion Transporter 1 Gene in the Kidney | American Journal of Physiology-Renal Physiology | 10.1152/ajprenal.00017.2007 | basic experiment |
| 125 | Park, J., J. Pyee and H. Park | 2014 | Pinosylvin at a High Concentration Induces Ampk-Mediated Autophagy for Preventing Necrosis in Bovine Aortic Endothelial Cells | Canadian Journal of Physiology and Pharmacology | 10.1139/cjpp-2014-0271 | basic experiment |
| 126 | Parsons, S. P. and J. D. Huizinga | 2015 | Effects of Gap Junction Inhibition on Contraction Waves in the Murine Small Intestine in Relation to Coupled Oscillator Theory | American Journal of Physiology-Gastrointestinal and Liver Physiology | 10.1152/ajpgi.00338.2014 | basic experiment |
| 127 | Peinkofer, G., J. Hescheler and M. Halbach | 2017 | Murine Short Axis Ventricular Heart Slices for Electrophysiological Studies | Jove-Journal of Visualized Experiments | 10.3791/55725 | basic experiment |
| 128 | Penn, A. C., S. R. Williams and I. H. Greger | 2008 | Gating Motions Underlie Ampa Receptor Secretion from the Endoplasmic Reticulum | Embo Journal | 10.1038/emboj.2008.222 | basic experiment |
| 129 | Peracchia, C. and L. L. Peracchia | 2005 | Inversion of Both Gating Polarity and Co<Sub>2</Sub> Sensitivity of Voltage Gating with D3n Mutation of Cx50 | American Journal of Physiology-Cell Physiology | 10.1152/ajpcell.00348.2004 | basic experiment |
| 130 | Popa, T., C. Hubsch, P. James, et al. | 2018 | Abnormal Cerebellar Processing of the Neck Proprioceptive Information Drives Dysfunctions in Cervical Dystonia | Scientific Reports | 10.1038/s41598-018-20510-1 | basic experiment |
| 131 | Porat, Z., I. Yaron, B. Z. Katz, et al. | 2011 | Shear Flow-Induced Formation of Tubular Cell Protrusions in Multiple Myeloma Cells | Journal of Cellular Physiology | 10.1002/jcp.22680 | basic experiment |
| 132 | Potse, M., A. Vinet, T. Opthof, et al. | 2009 | Validation of a Simple Model for the Morphology of the T Wave in Unipolar Electrograms | American Journal of Physiology-Heart and Circulatory Physiology | 10.1152/ajpheart.00064.2009 | basic experiment |
| 133 | Praetorius, H. A., J. Frokiær and J. Leipziger | 2005 | Transepithelial Pressure Pulses Induce Nucleotide Release in Polarized Mdck Cells | American Journal of Physiology-Renal Physiology | 10.1152/ajprenal.00238.2004 | basic experiment |
| 134 | Qu, H., L. Cui, J. Rickers-Haunerland, et al. | 2007 | Fatty Acid-Dependent Expression of the Muscle Fabp Gene - Comparative Analysis of Gene Control in Functionally Related, but Evolutionary Distant Animal Systems | Molecular and Cellular Biochemistry | 10.1007/s11010-005-9036-z | basic experiment |
| 135 | Quiñonez, M. and M. DiFranco | 2000 | Novel Inverted Triple Grease-Gap Isolation Chamber for Electrophysiological and Calcium Release Studies in Skeletal Muscle Fibers | Japanese Journal of Physiology | 10.2170/jjphysiol.50.457 | basic experiment |
| 136 | Ren, M. Q., Y. T. Zhou, H. X. Chen, et al. | 2018 | Quantitative Analysis of Multiple Elements in Healthy and Remodeled Epithelium from Human Upper Airway Mucosa Using Nuclear Microscopy | Allergy | 10.1111/all.13329 | basic experiment |
| 137 | Rescigno, M., V. Piguet, B. Valzasina, et al. | 2000 | Fas Engagement Induces the Maturation of Dendritic Cells (Dcs), the Release of Interleukin (Il)-1β, and the Production of Interferon Γ in the Absence of Il-12 During Dc-T Cell Cognate Interaction:: A New Role for Fas Ligand in Inflammatory Responses | Journal of Experimental Medicine | 10.1084/jem.192.11.1661 | basic experiment |
| 138 | Ridgway, L. D., E. Y. Kim and S. E. Dryer | 2009 | Magi-1 Interacts with Slo1 Channel Proteins and Suppresses Slo1 Expression on the Cell Surface | American Journal of Physiology-Cell Physiology | 10.1152/ajpcell.00073.2009 | basic experiment |
| 139 | Rodríguez-Arias, M., A. Castillo, M. Daza-Losada, et al. | 2009 | Effects of Extended Cocaine Conditioning in the Reinstatement of Place Preference | Physiology & Behavior | 10.1016/j.physbeh.2008.12.011 | basic experiment |
| 140 | Roppongi, R. T., K. P. Champagne-Jorgensen and T. J. Siddiqui | 2017 | Low-Density Primary Hippocampal Neuron Culture | Jove-Journal of Visualized Experiments | 10.3791/55000 | basic experiment |
| 141 | Rosen, R., J. M. Garza and S. Nurko | 2020 | Functional Luminal Imaging Probe Assessment in Postfundoplication Patients Changes Management Beyond Manometry | Journal of Pediatric Gastroenterology and Nutrition | 10.1097/mpg.0000000000002658 | basic experiment |
| 142 | Rosen, S. P., S. M. Abdelhalim, C. A. Jones, et al. | 2018 | Effect of Body Position on Pharyngeal Swallowing Pressures Using High-Resolution Manometry | Dysphagia | 10.1007/s00455-017-9866-3 | basic experiment |
| 143 | Rossi, A. C., M. Pluijmert, P. H. M. Bovendeerd, et al. | 2015 | Assessment and Comparison of Left Ventricular Shear in Normal and Situs Inversus Totalis Hearts by Means of Magnetic Resonance Tagging | American Journal of Physiology-Heart and Circulatory Physiology | 10.1152/ajpheart.00502.2014 | basic experiment |
| 144 | Saibene, A. M., G. Felisati, C. Pipolo, et al. | 2020 | Partial Preservation of the Inferior Turbinate in Endoscopic Medial Maxillectomy: A Computational Fluid Dynamics Study | American Journal of Rhinology & Allergy | 10.1177/1945892420902005 | basic experiment |
| 145 | Saito, H., T. Terada, J. Shimakura, et al. | 2008 | Regulatory Mechanism Governing the Diurnal Rhythm of Intestinal H<Sup>+</Sup>/Peptide Cotransporter 1 (Pept1) | American Journal of Physiology-Gastrointestinal and Liver Physiology | 10.1152/ajpgi.90317.2008 | basic experiment |
| 146 | Sakanaka, M., S. Nakakawaji, S. Nakajima, et al. | 2018 | A Transposon Mutagenesis System for <I>Bifidobacterium Longum </I>Subsp <I>Longum</I> Based on an Is<I>3</I> Family Insertion Sequence, Is<I>Blo11</I> | Applied and Environmental Microbiology | 10.1128/aem.00824-18 | basic experiment |
| 147 | Sardanyés, J., A. Bonforti, N. Conde, et al. | 2015 | Computational Implementation of a Tunable Multicellular Memory Circuit for Engineered Eukaryotic Consortia | Frontiers in Physiology | 10.3389/fphys.2015.00281 | basic experiment |
| 148 | Sardella, D., A. M. Kristensen, L. Bordoni, et al. | 2023 | Serial Intravital 2-Photon Microscopy and Analysis of the Kidney Using Upright Microscopes | Frontiers in Physiology | 10.3389/fphys.2023.1176409 | basic experiment |
| 149 | Schmidt, E., J. Gutberlet, D. Siegmund, et al. | 2009 | Apoptosis Is Not Required for Acantholysis in Pemphigus Vulgaris | American Journal of Physiology-Cell Physiology | 10.1152/ajpcell.00161.2008 | basic experiment |
| 150 | Schroder, E. A., D. E. Burgess, C. L. Manning, et al. | 2014 | Light Phase-Restricted Feeding Slows Basal Heart Rate to Exaggerate the Type-3 Long Qt Syndrome Phenotype in Mice | American Journal of Physiology-Heart and Circulatory Physiology | 10.1152/ajpheart.00341.2014 | basic experiment |
| 151 | Selli, A. L., A. K. Kuzmiszyn, N. Smaglyukova, et al. | 2021 | Treatment of Cardiovascular Dysfunction with Pde5-Inhibitors - Temperature Dependent Effects on Transport and Metabolism of Camp and Cgmp | Frontiers in Physiology | 10.3389/fphys.2021.695779 | basic experiment |
| 152 | Sempou, E., O. A. Lakhani, S. Amalraj, et al. | 2018 | Candidate Heterotaxy Gene Fgfr4 Is Essential for Patterning of the Left-Right Organizer in Xenopus | Frontiers in Physiology | 10.3389/fphys.2018.01705 | basic experiment |
| 153 | Seo, J. B., M. Moody and D. S. Koh | 2014 | Epithelial Monolayer Culture System for Real-Time Single-Cell Analyses | Physiological Reports | 10.14814/phy2.12002 | basic experiment |
| 154 | Sethuraman, V., B. S. Shu, G. F. Cui, et al. | 2017 | Curcumin Induces Autophagic Cell Death in <I>Spodoptera Frugiperda</I> Cells | Pesticide Biochemistry and Physiology | 10.1016/j.pestbp.2017.05.004 | basic experiment |
| 155 | Sezgunsay, E. and T. Basak | 2020 | Is Moulage Effective in Improving Clinical Skills of Nursing Students for the Assessment of Pressure Injury? | Nurse Education Today | 10.1016/j.nedt.2020.104572 | basic experiment |
| 156 | Sidorov, V. Y., P. C. Samson, T. N. Sidorova, et al. | 2017 | I-Wire Heart-on-a -Chip I: Three-Dimensional Cardiac Tissue Constructs for Physiology and Pharmacology | Acta Biomaterialia | 10.1016/j.actbio.2016.11.009 | basic experiment |
| 157 | Simonson, S. R. | 2014 | Making Students Do the Thinking: Team-Based Learning in a Laboratory Course | Advances in Physiology Education | 10.1152/advan.00108.2013 | basic experiment |
| 158 | Siran, R., A. H. Ahmad, C. B. A. Aziz, et al. | 2014 | Rem Sleep Deprivation Induces Changes of Down Regulatory Antagonist Modulator (Dream) Expression in the Ventrobasal Thalamic Nuclei of Sprague-Dawley Rats | Journal of Physiology and Biochemistry | 10.1007/s13105-014-0356-x | basic experiment |
| 159 | Song, C., S. Choi, K. B. Oh, et al. | 2020 | Suppression of Trpm7 Enhances Trail-Induced Apoptosis in Triple-Negative Breast Cancer Cells | Journal of Cellular Physiology | 10.1002/jcp.29820 | basic experiment |
| 160 | Sorensen, A., M. Sinding, D. A. Peters, et al. | 2015 | Placental Oxygen Transport Estimated by the Hyperoxic Placental Bold Mri Response | Physiological Reports | 10.14814/phy2.12582 | basic experiment |
| 161 | Sové, R. J., S. Milkovich, H. N. Nikolov, et al. | 2021 | Localized Oxygen Exchange Platform for Intravital Video Microscopy Investigations of Microvascular Oxygen Regulation | Frontiers in Physiology | 10.3389/fphys.2021.654928 | basic experiment |
| 162 | Srinivas, S. P., J. A. Bonanno, E. Larivière, et al. | 2003 | Measurement of Rapid Changes in Cell Volume by Forward Light Scattering | Pflugers Archiv-European Journal of Physiology | 10.1007/s00424-003-1145-5 | basic experiment |
| 163 | Stoyanova, R., J. J. Upson, C. Patriotis, et al. | 2004 | Use of Rna Amplification in the Optimal Characterization of Global Gene Expression Using Cdna Microarrays | Journal of Cellular Physiology | 10.1002/jcp.20074 | basic experiment |
| 164 | Takeda, K., H. Kasai, H. Tajima, et al. | 2023 | Mixed-Methods Education of Mechanical Ventilation for Residents in the Era of the Covid-19 Pandemic: Preliminary Interventional Study | Plos One | 10.1371/journal.pone.0287925 | basic experiment |
| 165 | Tan, J. Y., X. Wang, K. Devadas, et al. | 2013 | Some Mechanisms of Flip Expression in Inhibition of Hiv-1 Replication in Jurkat Cells, Cd4+T Cells and Pbmcs | Journal of Cellular Physiology | 10.1002/jcp.24397 | basic experiment |
| 166 | Tawfik, H., S. Puza, R. Seemann, et al. | 2020 | Transport Properties of Gramicidin a Ion Channel in a Free-Standing Lipid Bilayer Filled with Oil Inclusions | Frontiers in Cell and Developmental Biology | 10.3389/fcell.2020.531229 | basic experiment |
| 167 | Teitelbaum, E. N., L. Boris, F. O. Arafat, et al. | 2013 | Comparison of Esophagogastric Junction Distensibility Changes During Poem and Heller Myotomy Using Intraoperative Flip | Surgical Endoscopy and Other Interventional Techniques | 10.1007/s00464-013-3121-2 | basic experiment |
| 168 | Teitelbaum, E. N., N. J. Soper, J. E. Pandolfino, et al. | 2014 | An Extended Proximal Esophageal Myotomy Is Necessary to Normalize Egj Distensibility During Heller Myotomy for Achalasia, but Not Poem | Surgical Endoscopy and Other Interventional Techniques | 10.1007/s00464-014-3563-1 | basic experiment |
| 169 | Teitelbaum, E. N., N. J. Soper, J. E. Pandolfino, et al. | 2015 | Esophagogastric Junction Distensibility Measurements During Heller Myotomy and Poem for Achalasia Predict Postoperative Symptomatic Outcomes | Surgical Endoscopy and Other Interventional Techniques | 10.1007/s00464-014-3733-1 | basic experiment |
| 170 | Teitelbaum, E. N., J. M. Sternbach, R. El Khoury, et al. | 2016 | The Effect of Incremental Distal Gastric Myotomy Lengths on Egj Distensibility During Poem for Achalasia | Surgical Endoscopy and Other Interventional Techniques | 10.1007/s00464-015-4269-8 | basic experiment |
| 171 | Theron, A. E., E. M. Nolte, L. Lafanechère, et al. | 2013 | Molecular Crosstalk between Apoptosis and Autophagy Induced by a Novel 2-Methoxyestradiol Analogue in Cervical Adenocarcinoma Cells | Cancer Cell International | 10.1186/1475-2867-13-87 | basic experiment |
| 172 | Thompson, C., K. Keck and A. Hielscher | 2017 | Isolation of Intact, Whole Mouse Mammary Glands for Analysis of Extracellular Matrix Expression and Gland Morphology | Jove-Journal of Visualized Experiments | 10.3791/56512 | basic experiment |
| 173 | Trägardh, E., M. Claesson, G. S. Wagner, et al. | 2007 | Detection of Acute Myocardial Infarction Using the 12-Lead Ecg Plus Inverted Leads Versus the 16-Lead Ecg (with Additional Posterior and Right-Sided Chest Electrodes) | Clinical Physiology and Functional Imaging | 10.1111/j.1475-097X.2007.00761.x | basic experiment |
| 174 | Trepat, X., M. Grabulosa, F. Puig, et al. | 2004 | Viscoelasticity of Human Alveolar Epithelial Cells Subjected to Stretch | American Journal of Physiology-Lung Cellular and Molecular Physiology | 10.1152/ajplung.00077.2004 | basic experiment |
| 175 | Trepat, X., F. Puig, N. Gavara, et al. | 2006 | Effect of Stretch on Structural Integrity and Micromechanics of Human Alveolar Epithelial Cell Monolayers Exposed to Thrombin | American Journal of Physiology-Lung Cellular and Molecular Physiology | 10.1152/ajplung.00436.2005 | basic experiment |
| 176 | Triggiani, A. I., A. Valenzano, M. A. P. Ciliberti, et al. | 2017 | Heart Rate Variability Is Reduced in Underweight and Overweight Healthy Adult Women | Clinical Physiology and Functional Imaging | 10.1111/cpf.12281 | basic experiment |
| 177 | Tung, T. C., K. Oshima, G. Cui, et al. | 2003 | Dual Upregulation of Fas and Bax Promotes Alloreactive T Cell Apoptosis in Il-10 Gene Targeting of Cardiac Allografts | American Journal of Physiology-Heart and Circulatory Physiology | 10.1152/ajpheart.00976.2002 | basic experiment |
| 178 | Ulfig, A., V. Bader, M. Varatnitskaya, et al. | 2021 | Hypochlorous Acid-Modified Human Serum Albumin Suppresses Mhc Class Ii-Dependent Antigen Presentation in Pro-Inflammatory Macrophages | Redox Biology | 10.1016/j.redox.2021.101981 | basic experiment |
| 179 | Ung, C. Y., S. H. Lam, X. Zhang, et al. | 2011 | Existence of Inverted Profile in Chemically Responsive Molecular Pathways in the Zebrafish Liver | Plos One | 10.1371/journal.pone.0027819 | basic experiment |
| 180 | VaezMousavi, S. M., R. J. Barry and A. R. Clarke | 2009 | Individual Differences in Task-Related Activation and Performance | Physiology & Behavior | 10.1016/j.physbeh.2009.06.007 | basic experiment |
| 181 | van Zyl, F. J., J. Marais, M. Nieuwoudt, et al. | 2019 | Computer Simulation and Physical Phantom Models for Estimating the Dielectric Properties of Rhinoceros Tissue | Plos One | 10.1371/journal.pone.0216595 | basic experiment |
| 182 | Venza, I., M. Visalli, R. Oteri, et al. | 2014 | Class I-Specific Histone Deacetylase Inhibitor Ms-275 Overrides Trail-Resistance in Melanoma Cells by Downregulating C-Flip | International Immunopharmacology | 10.1016/j.intimp.2014.05.024 | basic experiment |
| 183 | Venza, M., M. Visalli, R. Oteri, et al. | 2015 | The Overriding of Trail Resistance by the Histone Deacetylase Inhibitor Ms-275 Involves C-Myc up-Regulation in Cutaneous, Uveal, and Mucosal Melanoma | International Immunopharmacology | 10.1016/j.intimp.2015.06.024 | basic experiment |
| 184 | Vijeepallam, K., V. Pandy, T. Kunasegaran, et al. | 2016 | <I>Mitragyna Speciosa</I> Leaf Extract Exhibits Antipsychotic-Like Effect with the Potential to Alleviate Positive and Negative Symptoms of Psychosis in Mice | Frontiers in Pharmacology | 10.3389/fphar.2016.00464 | basic experiment |
| 185 | Vo, A. N., S. Kundu, C. Strong, et al. | 2022 | Enhancement of Neuroglial Extracellular Matrix Formation and Physiological Activity of Dopaminergic Neural Cocultures by Macromolecular Crowding | Cells | 10.3390/cells11142131 | basic experiment |
| 186 | Walker, C. P., J. B. Buse and F. Frohlich | 2021 | Experimental Increase of Blood Glucose Alters Resting State Eeg Measures of Excitation-Inhibition Balance | Experimental Physiology | 10.1113/ep089211 | basic experiment |
| 187 | Wang, H. F., Z. G. Liu, Y. Wang, et al. | 2020 | Genome-Wide Differential DNA Methylation in Reproductive, Morphological, and Visual System Differences between Queen Bee and Worker Bee (<I>Apis Mellifera</I>) | Frontiers in Genetics | 10.3389/fgene.2020.00770 | basic experiment |
| 188 | Wang, H. Y., F. Li, W. R. Ban, et al. | 2021 | Human Bone Marrow Mesenchymal Stromal Cell-Derived Extracellular Vesicles Promote Proliferation of Degenerated Nucleus Pulposus Cells and the Synthesis of Extracellular Matrix through the Sox4/Wnt/Β-Catenin Axis | Frontiers in Physiology | 10.3389/fphys.2021.723220 | basic experiment |
| 189 | Wang, R. D., F. J. Kong, H. Z. Wu, et al. | 2020 | Complete Genome Sequence of High-Yield Strain <I>S. Lincolnensis</I> B48 and Identification of Crucial Mutations Contributing to Lincomycin Overproduction | Synthetic and Systems Biotechnology | 10.1016/j.synbio.2020.03.001 | basic experiment |
| 190 | Wang, Y., L. L. Zhang, X. M. Xi, et al. | 2021 | The Association between Etiologies and Mortality in Acute Respiratory Distress Syndrome: A Multicenter Observational Cohort Study | Frontiers in Medicine | 10.3389/fmed.2021.739596 | basic experiment |
| 191 | Wang, Y. L., J. Tang, V. P. Vimal, et al. | 2022 | Crash Prediction Using Deep Learning in a Disorienting Spaceflight Analog Balancing Task | Frontiers in Physiology | 10.3389/fphys.2022.806357 | basic experiment |
| 192 | Wayner, M. J., H. A. Tracy, D. L. Armstrong, et al. | 2000 | Air Righting: Role of the Nmda Receptor Channel and Hippocampal Ltp | Physiology & Behavior | 10.1016/s0031-9384(00)00208-0 | basic experiment |
| 193 | Weber, T., I. Lang, R. Zweiker, et al. | 2016 | Hypertension and Coronary Artery Disease: Epidemiology, Physiology, Effects of Treatment, and Recommendations | Wiener Klinische Wochenschrift | 10.1007/s00508-016-0998-5 | basic experiment |
| 194 | Wei, S. C., X. Y. Shen, Z. D. Gong, et al. | 2017 | Fshr and Lhr Expression and Signaling as Well as Maturation and Apoptosis of Cumulus-Oocyte Complexes Following Treatment with Fsh Receptor Binding Inhibitor in Sheep | Cellular Physiology and Biochemistry | 10.1159/000480650 | basic experiment |
| 195 | Wesseling, M. C., L. Wagner-Britz, D. B. Nguyen, et al. | 2016 | Novel Insights in the Regulation of Phosphatidylserine Exposure in Human Red Blood Cells | Cellular Physiology and Biochemistry | 10.1159/000447891 | basic experiment |
| 196 | Wilding, T. J., K. Chen and J. E. Huettner | 2010 | Fatty Acid Modulation and Polyamine Block of Gluk2 Kainate Receptors Analyzed by Scanning Mutagenesis | Journal of General Physiology | 10.1085/jgp.201010442 | basic experiment |
| 197 | Williams, S. R., D. Zies, S. V. Mullegama, et al. | 2012 | Smith-Magenis Syndrome Results in Disruption of <I>Clock</I> Gene Transcription and Reveals an Integral Role for Rai1 in the Maintenance of Circadian Rhythmicity | American Journal of Human Genetics | 10.1016/j.ajhg.2012.04.013 | basic experiment |
| 198 | Wu, H., S. Qu, C. Y. Lu, et al. | 2012 | Genomic and Transcriptomic Insights into the Thermo-Regulated Biosynthesis of Validamycin in <I>Streptomyces Hygroscopicus</I> 5008 | Bmc Genomics | 10.1186/1471-2164-13-337 | basic experiment |
| 199 | Xin, H. R., F. Deng, M. Y. Zhou, et al. | 2021 | A Multi-Tissue Multi-Omics Analysis Reveals Distinct Kineztics in Entrainment of Diurnal Transcriptomes by Inverted Feeding | Iscience | 10.1016/j.isci.2021.102335 | basic experiment |
| 200 | Yang, B. F., C. Xiao, H. Li, et al. | 2007 | Resistance to Fas-Mediated Apoptosis in Malignant Tumours Is Rescued by Kn-93 and Cisplatin Via Downregulation of C-Flip Expression and Phosphorylation | Clinical and Experimental Pharmacology and Physiology | 10.1111/j.1440-1681.2007.04711.x | basic experiment |
| 201 | Yin, J., B. Ni, W. G. Liao, et al. | 2018 | Hypoxia-Induced Apoptosis of Mouse Spermatocytes Is Mediated by Hif-1 through a Death Receptor Pathway and a Mitochondrial Pathway | Journal of Cellular Physiology | 10.1002/jcp.25974 | basic experiment |
| 202 | Yu, H., G. Liu, G. Zhao, et al. | 2018 | Identification of a Conserved DNA Sulfur Recognition Domain by Characterizing the Phosphorothioate-Specific Endonuclease Sprmcra from <I>Streptomyces Pristinaespiralis</I> | Molecular Microbiology | 10.1111/mmi.14118 | basic experiment |
| 203 | Zhang, S. Q., T. al-Maghout, Y. T. Zhou, et al. | 2016 | Role of Dicer Enzyme in the Regulation of Store Operated Calcium Entry (Soce) in Cd4<Sup>+</Sup> T Cells | Cellular Physiology and Biochemistry | 10.1159/000447840 | basic experiment |
| 204 | Zheng, H. F., B. T. Drumm, M. H. Zhu, et al. | 2020 | Na<Sup>+</Sup>/Ca<Sup>2+</Sup> Exchange and Pacemaker Activity of Interstitial Cells of Cajal | Frontiers in Physiology | 10.3389/fphys.2020.00230 | basic experiment |
| 205 | Zhu, W. D., T. L. Voelker, Z. Varga, et al. | 2017 | Mechanisms of Noncovalent Β Subunit Regulation of Na<Sub>V</Sub> Channel Gating | Journal of General Physiology | 10.1085/jgp.201711802 | basic experiment |
| 206 | Zollinger, M., F. Degache, G. Currat, et al. | 2016 | External Mechanical Work and Pendular Energy Transduction of Overground and Treadmill Walking in Adolescents with Unilateral Cerebral Palsy | Frontiers in Physiology | 10.3359/fphys.2016.00121 | basic experiment |
| 207 | Bergemann, A. D., R. Lucito, J. M. Willey, et al. | 2021 | Using the "Hallmarks of Cancer" as a Framework for Medical Students and Clinicians to Understand Oncogenesis | Advances in Physiology Education | 10.1152/advan.00092.2020 | not flipped classroom |
| 208 | Bingen, H. M., B. Tveit, R. J. Krumsvik, et al. | 2019 | Nursing Students' Experiences with the Use of a Student Response System When Learning Physiology | Nordic Journal of Digital Literacy | 10.18261/issn.1891-943x-2019-01-02-04 | not flipped classroom |
| 209 | Chandran, D. S., S. P. Muthukrishnan, S. M. Barman, et al. | 2020 | Iups Physiology Education Workshop Series in India: Organizational Mechanics, Outcomes, and Lessons | Advances in Physiology Education | 10.1152/advan.00128.2020 | not flipped classroom |
| 210 | Goodman, B. E., D. S. Martin and J. L. Williams | 2002 | Teaching Human Cardiovascular and Respiratory Physiology with the Station Method | Advances in Physiology Education | 10.1152/advan.00034.2001 | not flipped classroom |
| 211 | Halpin, P. A. and C. Gopalan | 2021 | Using Dramatizations to Teach Cell Signaling Enhances Learning and Improves Students' Confidence in the Concept | Advances in Physiology Education | 10.1152/advan.00177.2020 | not flipped classroom |
| 212 | Islam, M. A., G. Sabnis and F. Farris | 2017 | The Trilayer Approach of Teaching Physiology, Pathophysiology, and Pharmacology Concepts in a First-Year Pharmacy Course: The Tlat Model | Advances in Physiology Education | 10.1152/advan.00047.2017 | not flipped classroom |
| 213 | Anderson, L. C. and T. Jacobson | 2023 | Providing the Choice of in-Person or Videoconference Attendance in a Clinical Physiology Course May Harm Learning Outcomes for the Entire Cohort | Advances in Physiology Education | 10.1152/advan.00160.2022 | not basic physiology |
| 214 | Bahri, A., I. S. Idris, H. Muis, et al. | 2021 | Blended Learning Integrated with Innovative Learning Strategy to Improve Self-Regulated Learning | International Journal of Instruction | 10.29333/iji.2021.14147a | not basic physiology |
| 215 | Ding, C., Q. Wang, J. L. Zou, et al. | 2021 | Implementation of Flipped Classroom Combined with Case- and Team-Based Learning in Residency Training | Advances in Physiology Education | 10.1152/advan.00022.2020 | not basic physiology |
| 216 | French, H., M. Gray, M. Gillam-Krakauer, et al. | 2018 | Flipping the Classroom: A National Pilot Curriculum for Physiology in Neonatal-Perinatal Medicine | Journal of Perinatology | 10.1038/s41372-018-0185-9 | not basic physiology |
| 217 | Acharya, S., S. Halder, D. A. Carlson, et al. | 2021 | Estimation of Mechanical Work Done to Open the Esophagogastric Junction Using Functional Lumen Imaging Probe Panometry | American Journal of Physiology-Gastrointestinal and Liver Physiology | 10.1152/ajpgi.00032.2021 | not human subjects |
| 218 | Acharya, S., S. Halder, D. A. Carlson, et al. | 2021 | Assessment of Esophageal Body Peristaltic Work Using Functional Lumen Imaging Probe Panometry | American Journal of Physiology-Gastrointestinal and Liver Physiology | 10.1152/ajpgi.00324.2020 | not human subjects |
| 219 | Akasaka, T., S. Hokimoto, D. Sueta, et al. | 2016 | Sex Differences in the Impact of Cyp2c19 Polymorphisms and Low-Grade Inflammation on Coronary Microvascular Disorder | American Journal of Physiology-Heart and Circulatory Physiology | 10.1152/ajpheart.00911.2015 | not human subjects |
| 220 | Akyazi, I. and E. Eraslan | 2014 | Transmission of Stress between Cagemates: A Study in Rats | Physiology & Behavior | 10.1016/j.physbeh.2013.10.006 | not human subjects |
| 221 | AlSabagh, A. T., S. Rao and W. M. Renno | 2023 | The Impact of Heat Therapy on Neuromuscular Function and Muscle Atrophy in Diabetic Rats | Frontiers in Physiology | 10.3389/fphys.2022.1039588 | not human subjects |
| 222 | Amreen, H. M. Ali, M. Ahmad, et al. | 2020 | Construction of a Nanosensor for Non-Invasive Imaging of Hydrogen Peroxide Levels in Living Cells | Biology-Basel | 10.3390/biology9120430 | not human subjects |
| 223 | Austen, J. M., J. A. Strickland and D. J. Sanderson | 2016 | Memory-Dependent Effects on Palatability in Mice | Physiology & Behavior | 10.1016/j.physbeh.2016.09.001 | not human subjects |
| 224 | Campos, L. A., R. Plehm, J. Cipolla-Neto, et al. | 2006 | Altered Circadian Rhythm Reentrainment to Light Phase Shifts in Rats with Low Levels of Brain Angiotensinogen | American Journal of Physiology-Regulatory Integrative and Comparative Physiology | 10.1152/ajpregu.00703.2005 | not human subjects |
| 225 | Duclos, M., C. Gouarne, C. Martin, et al. | 2004 | Effects of Corticosterone on Muscle Mitochondria Identifying Different Sensitivity to Glucocorticoids in Lewis and Fischer Rats | American Journal of Physiology-Endocrinology and Metabolism | 10.1152/ajpendo.00281.2003 | not human subjects |
| 226 | Ghasem, S., J. Majid and R. Shiva | 2013 | Embryo Developmental Capacity of Oocytes Fertilised by Sperm of Mouse Exposed to Forced Swimming Stress | Journal of the Pakistan Medical Association | | not human subjects |
| 227 | Gomide, J. M. C., R. C. de Menezes, L. G. Fernandes, et al. | 2013 | Increased Activity of the Renin-Angiotensin and Sympathetic Nervous Systems Is Required for Regulation of the Blood Pressure in Rats Fed a Low-Protein Diet | Experimental Physiology | 10.1113/expphysiol.2012.066712 | not human subjects |
| 228 | Graber, T. G., R. Maroto, C. S. Fry, et al. | 2021 | Measuring Exercise Capacity and Physical Function in Adult and Older Mice | Journals of Gerontology Series a-Biological Sciences and Medical Sciences | 10.1093/gerona/glaa205 | not human subjects |
| 229 | Huang, B. S., Q. S. Xie, X. C. Lu, et al. | 2016 | Glyt1 Inhibitor Nfps Exerts Neuroprotection Via Glyr Alphal Subunit in the Rat Model of Transient Focal Cerebral Ischaemia and Reperfusion | Cellular Physiology and Biochemistry | 10.1159/000445556 | not human subjects |
| 230 | Kharas, N., P. B. Yang, T. Robles, et al. | 2019 | Sex Differences in the Intensity of Cross-Sensitization between Methylphenidate and Amphetamine in Adolescent Rats | Physiology & Behavior | 10.1016/j.physbeh.2018.12.021 | not human subjects |
| 231 | Klonowski-Stumpe, H., R. Fischer, R. Reinehr, et al. | 2002 | Apoptosis in Activated Rat Pancreatic Stellate Cells | American Journal of Physiology-Gastrointestinal and Liver Physiology | 10.1152/ajpgi.00073.2002 | not human subjects |
| 232 | Landa, M. S., S. I. García, M. L. Schuman, et al. | 2007 | Knocking Down the Diencephalic Thyrotropin-Releasing Hormone Precursor Gene Normalizes Obesity-Induced Hypertension in the Rat | American Journal of Physiology-Endocrinology and Metabolism | 10.1152/ajpendo.00234.2006 | not human subjects |
| 233 | Laplante, M., W. T. Festuccia, G. Soucy, et al. | 2007 | Involvement of Adipose Tissues in the Early Hypolipidemic Action of Pparγ Agonism in the Rat | American Journal of Physiology-Regulatory Integrative and Comparative Physiology | 10.1152/ajpregu.00761.2006 | not human subjects |
| 234 | Lin, J. J., X. Y. Sun, X. F. Dai, et al. | 2022 | Integrated Proteomics and Metabolomics Analysis in Pregnant Rat Hippocampus after Circadian Rhythm Inversion | Frontiers in Physiology | 10.3389/fphys.2022.941585 | not human subjects |
| 235 | Liu, S. J. | 2007 | Inhibition of L-Type Ca<Sup>2+</Sup> Channel Current and Negative Inotropy Induced by Arachidonic Acid in Adult Rat Ventricular Myocytes | American Journal of Physiology-Cell Physiology | 10.1152/ajpcell.00284.2007 | not human subjects |
| 236 | Nastiuk, K. L., J. W. Kim, M. Mann, et al. | 2003 | Androgen Regulation of Flice-Like Inhibitory Protein Gene Expression in the Rat Prostate | Journal of Cellular Physiology | 10.1002/jcp.10283 | not human subjects |
| 237 | Nawi, A., K. L. Eu, A. N. A. Faris, et al. | 2020 | Lipid Peroxidation in the Descending Thoracic Aorta of Rats Deprived of Rem Sleep Using the Inverted Flowerpot Technique | Experimental Physiology | 10.1113/ep088667 | not human subjects |
| 238 | Ramsay, D. S., J. Seaman and K. J. Kaiyala | 2011 | Nitrous Oxide Causes a Regulated Hypothermia: Rats Select a Cooler Ambient Temperature While Becoming Hypothermic | Physiology & Behavior | 10.1016/j.physbeh.2010.12.018 | not human subjects |
| 239 | Salomonsson, M., F. Gustafsson, D. Andreasen, et al. | 2002 | Local Electric Stimulation Causes Conducted Calcium Response in Rat Interlobular Arteries | American Journal of Physiology-Renal Physiology | 10.1152/ajprenal.00247.2001 | not human subjects |
| 240 | Sandbak, T., L. M. Rimol, F. K. Jellestad, et al. | 2000 | Relating Acoustic Startle Reactivity and Plasticity to Alcohol Consumption in Male Wistar Rats | Physiology & Behavior | 10.1016/s0031-9384(99)00239-5 | not human subjects |
| 241 | Severin, M. J., R. V. Campagno, A. Brandoni, et al. | 2019 | Time Evolution of Methotrexate-Induced Kidney Injury: A Comparative Study between Different Biomarkers of Renal Damage in Rats | Clinical and Experimental Pharmacology and Physiology | 10.1111/1440-1681.13122 | not human subjects |
| 242 | Townsend, E. A. and K. B. Freeman | 2017 | Comparison of Cocaine Reinforcement in Lean and Obese Zucker Rats: Relative Potency and Reinstatement of Extinguished Operant Responding | Physiology & Behavior | 10.1016/j.physbeh.2016.12.016 | not human subjects |
| 243 | Woodruff, E. R., L. E. Chun, L. R. Hinds, et al. | 2016 | Diurnal Corticosterone Presence and Phase Modulate Clock Gene Expression in the Male Rat Prefrontal Cortex | Endocrinology | 10.1210/en.2015-1884 | not human subjects |
| 244 | Beason-Abmayr, B., D. R. Caprette and C. Gopalan | 2021 | Flipped Teaching Eased the Transition from Face-to-Face Teaching to Online Instruction During the Covid-19 Pandemic | Advances in Physiology Education | 10.1152/advan.00248.2020 | not physiology |
| 245 | DeRuisseau, L. R. | 2016 | The Flipped Classroom Allows for More Class Time Devoted to Critical Thinking | Advances in Physiology Education | 10.1152/advan.00033.2016 | not physiology |
| 246 | Falck, A., H. French, R. Dadiz, et al. | 2023 | Best Practices and Educator Strategies for Facilitating a Flipped Classroom in Graduate Medical Education | American Journal of Perinatology | 10.1055/s-0043-1772227 | not physiology |
| 247 | Feng, Y. H., B. Zhao, J. Zheng, et al. | 2022 | Online Flipped Classroom with Team-Based Learning Promoted Learning Activity in a Clinical Laboratory Immunology Class: Response to the Covid-19 Pandemic | Bmc Medical Education | 10.1186/s12909-022-03917-3 | not physiology |
| 248 | Gopalan, C., C. Butts-Wilmsmeyer and V. Moran | 2021 | Virtual Flipped Teaching During the Covid-19 Pandemic | Advances in Physiology Education | 10.1152/advan.00061.2021 | not physiology |
| 249 | Gopalan, C., A. Fentem and A. L. Rever | 2020 | The Refinement of Flipped Teaching Implementation to Include Retrieval Practice | Advances in Physiology Education | 10.1152/advan.00143.2019 | not physiology |
| 250 | Lax, N., J. Morris and B. J. Kolber | 2017 | A Partial Flip Classroom Exercise in a Large Introductory General Biology Course Increases Performance at Multiple Levels | Journal of Biological Education | 10.1080/00219266.2016.1257503 | not physiology |
| 251 | McLean, S., S. M. Attardi, L. Faden, et al. | 2016 | Flipped Classrooms and Student Learning: Not Just Surface Gains | Advances in Physiology Education | 10.1152/advan.00098.2015 | not physiology |
| 252 | Paralikar, S., C. J. Shah, A. Joshi, et al. | 2022 | Acquisition of Higher-Order Cognitive Skills (Hocs) Using the Flipped Classroom Model: A Quasi-Experimental Study | Cureus Journal of Medical Science | 10.7759/cureus.24249 | not physiology |
| 253 | H. Qutob | 2022 | Effect of flipped classroom approach in the teaching of a hematology course | PLoS One | 10.1371/journal.pone.0267096 | not physiology |
