# Supplementary material for: Inverted Classroom Teaching of Physiology in Basic Medical Education: Bibliometric Visual Analysis
Source: JMIR Med Educ. 2024 Jun 25;10:e52224. doi: 10.2196/52224 (PMC11217164; doi:10.2196/52224)
Supplement: Multimedia Appendix 4 [file mededu-v10-e52224-s004.docx]

Distribution of countries publishing papers related to inverted teaching in physiology.

| Count of publication | Year of first publication | Country |
| --- | --- | --- |
| 18 | 2013 | USA |
| 8 | 2019 | PEOPLES R CHINA |
| 3 | 2020 | AUSTRALIA |
| 2 | 2019 | NORWAY |
| 2 | 2020 | PAKISTAN |
| 2 | 2022 | INDIA |
| 1 | 2020 | BELGIUM |
| 1 | 2021 | BRAZIL |
| 1 | 2021 | OMAN |
| 1 | 2022 | GERMANY |
| 1 | 2020 | COLOMBIA |
| 1 | 2019 | JAPAN |
| 1 | 2023 | SAUDI ARABIA |
| 1 | 2020 | SWITZERLAND |
| 1 | 2020 | SPAIN |
