# Supplementary material for: Inverted Classroom Teaching of Physiology in Basic Medical Education: Bibliometric Visual Analysis
Source: JMIR Med Educ. 2024 Jun 25;10:e52224. doi: 10.2196/52224 (PMC11217164; doi:10.2196/52224)
Supplement: Multimedia Appendix 5 [file mededu-v10-e52224-s005.docx]

The top 12 institutions publishing papers on ICT in physiology.

| Count of publication | Centrality | Year of first publication | Institution |
| --- | --- | --- | --- |
| 5 | 0 | 2017 | Southern Illinois University Edwardsville |
| 5 | 0 | 2017 | Southern Illinois University System |
| 4 | 0.02 | 2018 | Duke University |
| 3 | 0.01 | 2020 | AUSTRALIA |
| 3 | 0 | 2021 | University of Washington Seattle |
| 3 | 0 | 2021 | University of Texas System |
| 3 | 0 | 2021 | Vanderbilt University |
| 3 | 0 | 2022 | Central South University |
| 3 | 0 | 2021 | University of Rochester |
| 3 | 0 | 2021 | University of Pennsylvania |
| 3 | 0 | 2021 | University of Texas Health Science Center at San Antonio |
| 3 | 0 | 2021 | Yale University |
| 3 | 0.01 | 2021 | Pennsylvania Medicine |
| 3 | 0 | 2021 | University of Washington |
| 3 | 0.02 | 2021 | Baylor College of Medicine |
