# Supplementary material for: Inverted Classroom Teaching of Physiology in Basic Medical Education: Bibliometric Visual Analysis
Source: JMIR Med Educ. 2024 Jun 25;10:e52224. doi: 10.2196/52224 (PMC11217164; doi:10.2196/52224)
Supplement: Multimedia Appendix 7 [file mededu-v10-e52224-s007.docx]

The most influential authors of ITP research

| Count of publication | Centrality | Year of first publication | Authors |
| --- | --- | --- | --- |
| 5 | 0.02 | 2017 | Gopalan, Chaya |
| 3 | 0.01 | 2021 | Carbajal, Melissa M |
| 3 | 0.01 | 2021 | Falck, Alison J |
| 3 | 0 | 2021 | Johnston, Lindsay C |
| 3 | 0.01 | 2022 | Feng, Dandan |
| 3 | 0.01 | 2022 | Luo, Ziqiang |
| 3 | 0.01 | 2021 | French, Heather |
| 3 | 0.01 | 2021 | Dadiz, Rita |
| 3 | 0 | 2021 | Izatt, Susan |
| 3 | 0.01 | 2021 | Gillam-krakauer, Maria |
