# Supplementary material for: Inverted Classroom Teaching of Physiology in Basic Medical Education: Bibliometric Visual Analysis
Source: JMIR Med Educ. 2024 Jun 25;10:e52224. doi: 10.2196/52224 (PMC11217164; doi:10.2196/52224)
Supplement: Multimedia Appendix 8 [file mededu-v10-e52224-s008.docx]

**Primary journals that publish research papers on ICT in physiology**

| Count of publication | Centrality | Year | Cited journal |
| --- | --- | --- | --- |
| 34 | 0.34 | 2013 | ADV PHYSIOL EDUC |
| 22 | 0.54 | 2013 | AM J PHARM EDUC |
| 21 | 0.14 | 2013 | ACAD MED |
| 13 | 0.25 | 2014 | CBE-LIFE SCI EDUC |
| 13 | 0.2 | 2015 | COMPUT EDUC |
| 12 | 0 | 2013 | MED TEACH |
| 12 | 0 | 2020 | BMC MED EDUC |
| 12 | 0.08 | 2018 | ANAT SCI EDUC |
| 11 | 0.03 | 2016 | MED EDUC |
| 8 | 0.16 | 2016 | ADV MED EDUC PRACT |
| 8 | 0.04 | 2015 | INTERNET HIGH EDUC |
| 8 | 0 | 2019 | NURS EDUC TODAY |
| 7 | 0.07 | 2020 | PLOS ONE |
| 7 | 0.15 | 2017 | ADV HEALTH SCI EDUC |
| 6 | 0.01 | 2016 | CHEM EDUC RES PRACT |
| 5 | 0.07 | 2015 | J NUTR EDUC BEHAV |
| 5 | 0.09 | 2019 | ACAD RADIOL |
| 5 | 0.27 | 2015 | ACT LEARN HIGH EDUC |
| 5 | 0.05 | 2019 | CLIN TEACH |
| 5 | 0 | 2019 | P NATL ACAD SCI USA |
| 5 | 0.04 | 2015 | J ECON EDUC |
| 4 | 0.01 | 2019 | IEEE T EDUC |
| 4 | 0.06 | 2017 | CURR PHARM TEACH LEA |
| 4 | 0.02 | 2022 | BMC NURS |
| 4 | 0.05 | 2019 | BRIT J EDUC TECHNOL |
| 3 | 0.02 | 2016 | NEW ENGL J MED |
| 3 | 0 | 2014 | J EDUC PSYCHOL |
| 3 | 0.04 | 2016 | EDUC PSYCHOL REV |
| 3 | 0.05 | 2019 | ACAD PSYCHIATR |
| 3 | 0 | 2020 | PSYCHOL BULL |
| 3 | 0.02 | 2019 | 6 INT S ENG ED |
| 3 | 0 | 2016 | INT J INNOV SCI MATH |
| 3 | 0.01 | 2014 | MIND SOC DEV HIGHER |
| 3 | 0.04 | 2021 | BMJ OPEN |
| 3 | 0.01 | 2014 | REV EDUC RES |
| 3 | 0.1 | 2020 | AM PSYCHOL |
| 3 | 0 | 2018 | FLIP YOUR CLASSROOM |
| 3 | 0 | 2015 | THEOR PRACT |
| 3 | 0.01 | 2019 | AM J SURG |
| 3 | 0 | 2020 | J PERS SOC PSYCHOL |
| 3 | 0.03 | 2015 | EDUC LEADERSHIP |
| 3 | 0.03 | 2017 | INT J TEACH LEARN HI |
| 3 | 0.01 | 2016 | BIOCHEM MOL BIOL EDU |
