# Supplementary material for: Inverted Classroom Teaching of Physiology in Basic Medical Education: Bibliometric Visual Analysis
Source: JMIR Med Educ. 2024 Jun 25;10:e52224. doi: 10.2196/52224 (PMC11217164; doi:10.2196/52224)
Supplement: Multimedia Appendix 9 [file mededu-v10-e52224-s009.docx]

**Information of the main clusters of keywords in inverted teaching in physiology.**

| Cluster | Size | Silhouette | Year | Top terms (LSI) | Top terms (long-likelihood ratio) | Terms (mutual information) |
| --- | --- | --- | --- | --- | --- | --- |
| 0 | 19 | 0.723 | 2018 | renal physiology; medical students; academic stress; team-based learning; student preparedness \| team-based learning; student preparedness; student performance; renal physiology; medical students | modified team-based learning (5.34, 0.05); flipped teaching (5.34, 0.05); gastrointestinal and renal physiology (5.34, 0.05); unflipped (5.34, 0.05); academic stress (5.34, 0.05) | modified team-based learning (0.12); flipped teaching (0.12); gastrointestinal and renal physiology (0.12); unflipped (0.12); academic stress (0.12) |
| 1 | 17 | 0.76 | 2019 | physiology education; student perceptions; student achievement; blended approach; social isolation \| online teaching; teaching model; social isolation; medical students; student achievement | online teaching (8.13, 0.005); physiology education (8.13, 0.005); engagement (4.01, 0.05); poll (4.01, 0.05); meaningful learning (4.01, 0.05) | engagement (0.29); poll (0.29); meaningful learning (0.29); human physiology (0.29); student achievement (0.29) |
| 2 | 17 | 0.812 | 2018 | medical education; dental education; learning resources; self-directed learning; student satisfaction \| student satisfaction; instruction approach; learning style; inverted classroom model; self-directed learning | medical education (8.96, 0.005); self-directed learning (4.41, 0.05); dental education (4.41, 0.05); instruction approach (4.41, 0.05); learning style (4.41, 0.05) | self-directed learning (0.22); dental education (0.22); instruction approach (0.22); learning style (0.22); inverted classroom model (0.22) |
| 3 | 17 | 0.873 | 2019 | dental students; biomedical science; autonomic nervous system; graduate medical education; small private online course \| graduate medical education; adult learning; curriculum design; dental students; autonomic nervous system | flipped classroom (5.43, 0.05); dental students (3.3, 0.1); biomedical science (3.3, 0.1); small private online course (3.3, 0.1); gender (3.3, 0.1) | dental students (0.47); biomedical science (0.47); small private online course (0.47); gender (0.47); adult learning (0.47) |
| 4 | 14 | 0.958 | 2017 | learning effectiveness; long-term influences; classroom teaching; medical students; physiology teaching \| physiology teaching; learning effectiveness; long-term influences; classroom teaching; medical students | PowerPoint (5.34, 0.05); flipped classroom teaching (5.34, 0.05); physiology teaching (5.34, 0.05); whiteboard (5.34, 0.05); learning effectiveness (5.34, 0.05) | PowerPoint (0.12); flipped classroom teaching (0.12); physiology teaching (0.12); whiteboard (0.12); learning effectiveness (0.12) |
| 5 | 13 | 0.937 | 2017 | collaboration; motivation; learning; student characteristics; blended learning \| student characteristics; blended learning; learning performance; motivation; learning | student characteristics (5.34, 0.05); face-to-face (5.34, 0.05); learning performance (5.34, 0.05); learning (5.34, 0.05); blended learning (5.34, 0.05) | student characteristics (0.12); face-to-face (0.12); learning performance (0.12); learning (0.12); blended learning (0.12) |
| 6 | 12 | 0.905 | 2018 | active learning; nursing education; learning strategies; learning objectives \| learning objectives; learning strategies; active learning; nursing education | active learning (8.96, 0.005); nursing education (8.96, 0.005); learning strategies (4.41, 0.05); preparation (4.41, 0.05); mastery (4.41, 0.05) | learning strategies (0.22); preparation (0.22); mastery (0.22); learning objectives (0.22); metacognition (0.22) |
| 7 | 9 | 0.849 | 2016 | all-digital training course; covid-19 pandemic; instructional design \| instructional design; all-digital training course; covid-19 pandemic | remote (5.34, 0.05); teaching of psychology (5.34, 0.05); instructional design (5.34, 0.05); laboratory (5.34, 0.05); covid-19 pandemic (5.34, 0.05) | remote (0.12); teaching of psychology (0.12); instructional design (0.12); laboratory (0.12); covid-19 pandemic (0.12) |
| 8 | 5 | 1 | 2022 | e-learning; flipped design; peer teaching; physiology laboratory course | physiology laboratory course (7.12, 0.01); peer teaching (7.12, 0.01); flipped design (7.12, 0.01); e-learning (3.4, 0.1); flipped classroom (0.91, 0.5) | physiology laboratory course (0.05); peer teaching (0.05); flipped design (0.05); flipped classroom (0.04); learning strategies (0.02) |
| 9 | 4 | 1 | 2021 | educational tool; flipped class; intestinal absorption; physiology; team-based learning; team; undergraduate | educational tool (5.87, 0.05); intestinal absorption (5.87, 0.05); team-based learning (5.87, 0.05); flipped class (5.87, 0.05); team (5.87, 0.05) | educational tool (0.09); intestinal absorption (0.09); team-based learning (0.09); flipped class (0.09); team (0.09) |
| 10 | 4 | 1 | 2020 | students;motivation;constructivism;dysfunction;patient;taught;learn | students (5.87, 0.05); dysfunction (5.87, 0.05); patient (5.87, 0.05); learn (5.87, 0.05); taught (5.87, 0.05) | students (0.09); dysfunction (0.09); patient (0.09); learn (0.09); taught (0.09) |
